# On the Role of PDZ Domain-Encoding Genes in Drosophila Border Cell Migration

**DOI:** 10.1534/g3.112.004093

**Published:** 2012-11-01

**Authors:** George Aranjuez, Elizabeth Kudlaty, Michelle S. Longworth, Jocelyn A. McDonald

**Affiliations:** * Department of Molecular Genetics, Lerner Research Institute, Cleveland Clinic, Cleveland, Ohio 44195; † Department of Genetics and Genome Sciences, School of Medicine, Case Western Reserve University, Cleveland, Ohio 44106; ‡ Program in Biological Sciences, Northwestern University, Evanston, Illinois 60208

**Keywords:** collective migration, PSD95/Dlg/ZO-1 (PDZ) domains, border cells, JAK/STAT, Drosophila

## Abstract

Cells often move as collective groups during normal embryonic development and wound healing, although the mechanisms governing this type of migration are poorly understood. The *Drosophila melanogaster* border cells migrate as a cluster during late oogenesis and serve as a powerful *in vivo* genetic model for collective cell migration. To discover new genes that participate in border cell migration, 64 out of 66 genes that encode PDZ domain-containing proteins were systematically targeted by *in vivo* RNAi knockdown. The PDZ domain is one of the largest families of protein-protein interaction domains found in eukaryotes. Proteins that contain PDZ domains participate in a variety of biological processes, including signal transduction and establishment of epithelial apical-basal polarity. Targeting PDZ proteins effectively assesses a larger number of genes via the protein complexes and pathways through which these proteins function. *par-6*, a known regulator of border cell migration, was a positive hit and thus validated the approach. Knockdown of 14 PDZ domain genes disrupted migration with multiple RNAi lines. The candidate genes have diverse predicted cellular functions and are anticipated to provide new insights into the mechanisms that control border cell movement. As a test of this concept, two genes that disrupted migration were characterized in more detail: *big bang* and the Dlg5 homolog *CG6509*. We present evidence that Big bang regulates JAK/STAT signaling, whereas Dlg5/CG6509 maintains cluster cohesion. Moreover, these results demonstrate that targeting a selected class of genes by RNAi can uncover novel regulators of collective cell migration.

Regulated cell movement is critical for embryonic development, adult wound healing, and normal immune system function. Determining how cells migrate during normal processes can help us better understand how misregulated cell migration contributes to pathologies such as tumor metastasis and inflammation. While some cells migrate singly, others move as small or large groups in a type of migration called collective migration (Friedl and Gilmour 2009). Cells migrate collectively during gastrulation in the embryo and in epithelial sheet migration during wound closure. Notably, this type of group migration has also been observed during tumor invasion and metastasis (Friedl and Gilmour 2009; Yilmaz and Christofori 2010; Friedl *et al.* 2012). Migrating cells display striking morphological changes induced by dynamic rearrangement of actin filaments and cell-substrate adhesions, which together provide the necessary force for movement (Ridley 2011). Cells migrating collectively further need to coordinate such individual cell motility to precisely modulate cell-cell adhesions and the cytoskeleton among cells in the group (Friedl and Gilmour 2009). Our current understanding of the mechanisms that regulate these and other aspects of collective cell migration in tissues is fairly limited. Therefore, we have turned to a genetically amenable model, the Drosophila border cells, to identify new genes and pathways that control collective cell migration.

Border cells migrate as a cohesive cluster of 6–10 cells during late oogenesis in a highly regulated process (Montell 2003). Border cells are first specified in the anterior follicle cell epithelium at early stage 9. The follicular epithelium is a monolayer of ∼600 cells that surrounds the germline-derived cells of the egg chamber, the basic subunit of the Drosophila ovary. The cytokine-like protein Unpaired (Upd) is secreted from a pair of non-migratory cells, the polar cells, to activate Janus kinase (JAK)/signal transducer activator of transcription (STAT) signaling in the surrounding follicle cells (Silver and Montell 2001; Beccari *et al.* 2002; Ghiglione *et al.* 2002; Xi *et al.* 2003; Silver *et al.* 2005). Cells expressing the highest levels of active JAK/STAT at the anterior end of the egg chamber become border cells. The border cells form a cluster around the polar cells and subsequently detach from the epithelium. Border cells then migrate over ∼150 µm distance through the germline-derived nurse cell layer to reach the oocyte (Figure 1A). Previous genetic screens identified multiple essential regulators of border cell migration, including the highly conserved steroid hormone receptor and receptor tyrosine kinase (RTK) signaling pathways (Liu and Montell 1999; Bai *et al.* 2000; Duchek and Rørth 2001; Duchek *et al.* 2001; Silver and Montell 2001; McDonald *et al.* 2003; Mathieu *et al.* 2007). However, none of the screens to date were performed to saturation and, therefore, may have missed critical genes. Moreover, despite the discovery of these and other signaling pathways, in many cases the specific downstream effectors that interpret these signals to produce specific cellular responses in border cells remain unknown.

**Figure 1:**
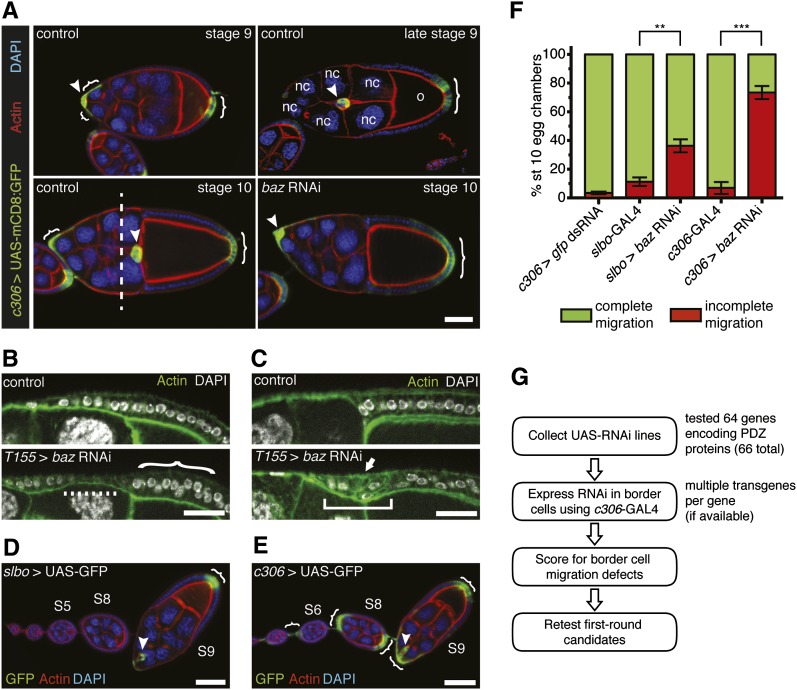
*In vivo* RNAi knockdown to identify PDZ domain-encoding genes required for border cell migration. (A) Control border cells (arrowheads) migrate between the nurse cells (nc) from stage 9 to 10 of oogenesis to reach the oocyte (o). Border cell clusters that have migrated past the dashed line (stage 10 control) are considered to have completed their migration. Border cells and follicle cells (brackets) express UAS-mCD8:GFP (green) driven by *c306*-GAL4 in egg chambers at the indicated stages; genotype is *c306-GAL4/*+; *UAS-mCD8:GFP/*+. Egg chambers were co-stained for actin (red) and DAPI (blue) to label cell membranes and nuclei, respectively. (Lower right panel) A stage 10 *c306-GAL4/*+; *UAS-mCD8:GFP/UAS-baz RNAi v2914* (*baz* RNAi) egg chamber in which border cells did not migrate. Scale bar is 20 μm. (B and C) Knockdown of *baz* in follicle cells (bottom panels) using the follicle cell driver *T155*-GAL4 disrupts the epithelium compared with control (top panels) at stage 9 (B) and stage 10 (C). Genotypes are *T155-GAL4/*+ (control) and *UAS-baz RNAi/*+; +/*T155-GAL4*. Scale bar is 20 μm. (B) *baz* RNAi follicle cell layer is thin (dashed line), and some nuclei are misaligned (bracket) compared with control. (C) *baz* RNAi follicle cells are multilayered (arrow) and fail to retract over the oocyte (square bracket) as in control. (D and E) Ovarioles showing GAL4 expression patterns in border cells (arrowheads) and follicle cells (brackets) as visualized by UAS-mCD8:GFP (green); stages are indicated. Egg chambers were co-stained for actin (red) and DAPI (blue). Scale bar is 50 μm. (D) *slbo*-GAL4 expression pattern (*slbo-GAL4*, *UAS-mCD8:GFP/*+). (E) *c306*-GAL4 expression pattern (*c306-GAL4/*+; *UAS-mCD8:GFP/*+). *c306*-GAL4 is also expressed in stalk cells, which connect egg chambers within the ovariole. (F) Quantification of migration in stage 10 egg chambers of the indicated genotypes, shown as the percentage with complete (green) or incomplete (red) border cell migration. Error bars represent SEM; n ≥ 50 egg chambers in each of three trials (**P < 0.01; ***P < 0.001; two-tailed unpaired *t*-test). (G) Outline of the scheme used to survey the role of PDZ genes in border cell migration. Anterior is to the left in this and all subsequent figures.

Correct establishment of cell polarity is critical for the motility of many types of cells, including border cells (Niewiadomska *et al.* 1999; Pinheiro and Montell 2004; Etienne-Manneville 2008; McDonald *et al.* 2008). Proteins that regulate epithelial polarity help orient migrating cells and promote motility of both single and collectively migrating cells by organizing the cellular membrane and cytoskeleton (Humbert *et al.* 2006; Etienne-Manneville 2008; Hidalgo-Carcedo *et al.* 2011). Moreover, many of these proteins themselves localize in a polarized manner within cells, typically at the cell cortex. Epithelial polarity proteins have also emerged as potential tumor suppressors (Etienne-Manneville 2008; Humbert *et al.* 2008; Royer and Lu 2011). A large number of polarity proteins implicated in cell migration, such as Par-3, Patj, and Dlg1, contain PSD95/Dlg/ZO-1 (PDZ) domains. The PDZ domain is a small globular module that functions as a protein-protein interaction domain (Harris and Lim 2001; Subbaiah *et al.* 2011). Specifically, PDZ domains bind to short PDZ-binding motifs (PBM) on target proteins that are mainly, although not exclusively, found at C-termini (Harris and Lim 2001; Subbaiah *et al.* 2011). PDZ domains can occur alone or as multiple copies and are often found in combination with other protein-protein interaction domains and/or catalytic domains. Proteins with PDZ domains typically mediate the formation of large multi-protein scaffolding complexes that bring molecular components into proximity with each other within the cell (Ranganathan and Ross 1997). PDZ domain-containing proteins regulate signaling, cytoskeletal dynamics, and cell adhesion in addition to polarity, all of which are important for cell motility. Moreover, the polarity PDZ proteins Bazooka (Baz; Par-3 homolog) and Par-6 organize the localization of membrane-associated proteins within the border cell cluster to promote migration (Pinheiro and Montell 2004). This raises the distinct possibility that other, unidentified PDZ domain complexes regulate the polarity and collective migration of border cells.

We sought to identify additional genes that participate in border cell migration using an RNAi knockdown approach. The recent availability of large collections of UAS-RNAi transgenic lines have made it possible to systematically analyze the roles of the majority of genes in the Drosophila genome for specific phenotypes (Dietzl *et al.* 2007; Ni *et al.* 2009). These lines are used to knock down gene function in a tissue- and temporal-specific manner using the GAL4/UAS system (Brand and Perrimon 1993; Perrimon *et al.* 2010). While multiple genome-wide *in vivo* RNAi screens have been performed (Cronin *et al.* 2009; Mummery-Widmer *et al.* 2009; Schnorrer *et al.* 2010), a substantial number of lines (>12,000) is needed to screen most of the genes in the genome at least once. Moreover, large-scale screens could miss more subtle phenotypes. Several recent studies targeted specific classes of proteins, for example Rab GTPase-activating proteins and kinases, to identify new regulators of cell migration (Simpson *et al.* 2008; Laflamme *et al.* 2012). In the present study, we specifically targeted by RNAi knockdown 64 out of 66 genes predicted to encode PDZ domain-containing proteins. We identified 14 high confidence and 17 additional PDZ domain-containing proteins whose knockdown inhibited border cell migration. We provide additional evidence that two genes, *big bang* and *CG6509*, regulate specific features of border cells. The genes identified here thus represent a group of conserved signaling pathways and/or intracellular protein complexes that may regulate other types of collectively migrating cells.

## Materials and Methods

### Drosophila genetics

All crosses were kept at 25° using standard protocols. *c306*-GAL4, *slbo*-GAL4, *hsp70*-GAL4 (*hs*-GAL4), *T155*-GAL4 (Bloomington Stock Center) and *tubulin*-GAL4 (from A. Page-McCaw) were used to drive UAS-RNAi expression. GAL4 lines were outcrossed to *w^1118^* and used as controls. *c96-GAL4*, *UAS-mCD8:GFP* (from A. Zhu) was used to study the *bbg* expression pattern (Gustafson and Boulianne 1996). The following lines for off-target genes were obtained from the Vienna Drosophila RNAi Center (VDRC) or Harvard Transgenic RNAi Project (TRiP) from the Bloomington Stock Center: UAS-*ERR* RNAi KK108422 (line v108349, VDRC); UAS-*Irbp* RNAi (line JF03273, TRiP); and UAS-*CG42724* GD4280 RNAi (line v30629, VDRC). Additional fly stocks from the Bloomington Stock Center were two insertions of UAS-CG6509.GFP (http://flybase.org/reports/FBrf0211100.html); *PsGef^Δ21^*, *FRT^19A^*; *PsGef^Δ55^*, *FRT^19A^*; UAS-mCD8::GFP; and UAS-GFP dsRNA.

### *In vivo* RNAi knockdown

RNAi lines were obtained from VDRC, TRiP, NIG-Fly, and the Bloomington Stock Center. Supporting Information, Table S1 provides the complete list of RNAi lines. Virgin *c306-GAL4*; *UAS-mCD8:GFP/CyO* flies were crossed with males from each UAS-RNAi line. Eight female progeny flies per cross were fattened by feeding with yeast paste for 20 hr at 29° prior to dissection to achieve maximal GAL4/UAS expression. The UAS-*baz* RNAi v2914 line (VDRC) and the UAS-GFP dsRNA (line 143) (http://flybase.org/reports/FBrf0191479.html) were used as positive and negative controls, respectively. The RNAi lines were tested in batches of 22 lines together with the controls in 24-well plates. Whole ovaries were dissected as described (McDonald and Montell 2005; Prasad *et al.* 2007). Ovaries were fixed with 4% formaldehyde in potassium phosphate buffer (pH 7.2) for 10 min and washed with potassium phosphate buffer. Fixed ovaries were manually dissociated in 80% glycerol. UAS-mCD8:GFP fluorescence was used to visualize border cells in dissociated ovaries. Analysis of border cell migration was performed with a Zeiss Stereo Discovery V8 epi-fluorescent stereomicroscope. Crosses were set up independently and retested as above to confirm first-round candidates.

### Quantitative RT-PCR analysis of gene expression

Virgin *hsp70*-GAL4 (*hs*-GAL4) flies were crossed to male UAS-RNAi flies. To express RNAi ubiquitously, adult female progeny were heat shocked for 1 hr, three times a day, at 37° for two days. Ovaries were dissected the following day. RNA was extracted from ovaries or adult female fly carcasses (ovaries removed) using Trizol (Invitrogen). To determine endogenous expression levels, RNA was extracted from 15 to 20 ovary pairs dissected from *hs*-GAL4/UAS-GFP dsRNA females. The endogenous expression levels of *rp49* and *tub84B* were measured as reference controls (see Table 2 for details). To determine RNAi knockdown, RNA was extracted from 10 to 15 *hs*-GAL4 > UAS-RNAi female fly carcasses. Note that ovaries were removed because the germline is potentially refractory to long double-stranded hairpin RNA knockdown (Ni *et al.* 2011). RNA was purified using the Qiagen RNAeasy Kit, followed by cDNA synthesis using the Taqman Reverse Transcription Kit (Applied Biosystems) and 1.5 μg of purified RNA. qRT-PCR was performed using the Roche Lightcycler 480 to run 15 μL reactions containing 0.5 µL cDNA, 0.5 μL 10 µM primer mix, and 7.5 μL of SYBR Green Master Mix (Roche). All qRT-PCR experiments were performed in triplicate on three separate biological samples. In each experiment, UAS-*baz* RNAi was used as the positive control, and UAS-dsRNA GFP (GFP RNAi) was used as the negative control. RNAi knockdown was calculated using the ΔΔC_T_ method using *rp49* gene expression for normalization.

The following primers were used: *baz* fwd, CAGGAGCTGCAGATGTCGGATG; *baz* rev, ctcgtgatcgccatcctccaaaag; *bbg* fwd, CAATCTCCACACAACGAGCTCCAC; *bbg* rev, ggagatgccgccaagcttagc; CG43955 fwd, GGCTTTGATAGCTGGGCGAGC; CG43955 rev, gggggccctgaacaagatgaag; *CG43707* fwd, GCGGATGGTCGAAACGATATTGCG; *CG43707* rev, cttcttgccggatgcattggcg; *CG6498* fwd, CCTGCTCCGGAAGATCTCCTATC; *CG6498* rev, ctggtaacggagcggtcagttc; *CG6509* fwd, CAGCATGATCAGAAGGCGATCCC; *CG6509* rev, cacctgcatccgttccagcag; *CG9588* fwd, GATGATCGTCTGTCGCGCCAG; *CG9588* rev, cgtggaggcgcagatcaacag; *cnk* fwd, CTCCAGCTGTATGGCCGTATG; *cnk* rev, ggcctacatcaacatcgccgag; *dlg1* fwd, CCCGGCGACAATGGCATCTATG; *dlg1* rev, ccagttcgtgcgttacgttctcc; *dysc* fwd, CTAGGATTGTATCACCGGGTCGC; *dysc* rev, gcgcgaccagcaaatcgatcatg; *Grip* fwd, CAGTCCCGACGAGGTGATGAC, *Grip* rev, cgggactccagtgtgctaaagc; *PICK1* fwd, GATTGGCATCAGCATTGGGGGTG; *PICK1* rev, cacgctcaccgaattcacagcc; *RhoGAP100F* fwd, CACGGGCTCAGCGATTTTCGTG; *RhoGAP100F* rev, cgcacgggtagtgctgaaattgg; *rp49* fwd, TACAGGCCCAAGATCGTGAAG; *rp49* rev, gacgcactctgttgtcgatacc; *scrib* fwd, CAATGAAATTGGCCGCCTGCCG; *scrib* rev, cgaacttgggtatcgggttcgaac; *sif* fwd, CAAAGTGGCGAGCTGCCCAATC; *sif* rev, caggttgttgagcagcgaggg; *Syn1* fwd, GAATTGGGCAGGGTGCCGTTC; *Syn1* rev, ctggaaacggacttcctggcc; *tub84B* fwd, GGCAAGGAGATCGTCGATCTGG; *tub84B* rev, gacgctccatcagcagcgag; *vari* fwd, CTCGTTCACGATGACCATGTCGAAG; *vari* rev, cataagattcagctccagacgcgc; *X11L* fwd, GCGTGTTGTTTCGGGCCAGATAC; *X11L* rev, cagtgctcggctgactttcgc.

### Immunostaining and microscopy

Ovarioles were dissected and fixed in 4% formaldehyde in 1× phosphate buffered saline (PBS) with 0.2% v/v Triton X-100 (PBT). Blocking, antibody incubations, and washes were done in PBT with 5 mg/mL BSA (PBT-BSA). The primary antibodies used were: 1:400 mouse anti-alpha-tubulin (DM1A, Sigma); 1:200 rabbit anti-aPKC-zeta (sc-216, Santa Cruz); 1:50 concentrated mouse anti-Dlg1 (4F3, Developmental Studies Hybridoma Bank; DSHB); 1:150 mouse anti-Singed (sn 7c, DSHB); 1:150 rat anti-E-cadherin (DCAD-2, DSHB); 1:10 mouse anti-Fasciclin III (FasIII; 7G10, DSHB); 1:500 rabbit anti-GFP (Life Technologies); 1:1000 rabbit anti-Stat92E (a gift from S. Hou); and 1:500 rabbit anti-Veli (a gift from E. Knust). Secondary antibodies conjugated to Alexa Fluor 488, Alexa Fluor 568, or Alexa Fluor 647 (Life Technologies) were used at 1:400 dilution. Actin was visualized with phalloidin conjugated to Alexa Fluor 568 or Alexa Fluor 647 (Life Technologies) used at 1:400 dilution. DAPI (0.05 μg/mL, Sigma) was used to visualize nuclei. Stained egg chambers were mounted on slides in Aqua-Poly/Mount (Polysciences, Inc.) and imaged with a Zeiss AxioImager Z1 epi-fluorescent compound microscope equipped with the ApoTome system and MRm CCD camera. Either a 20× Plan-Apochromat 0.75 numerical aperture (NA) or a 40× Plan-Neofluar 1.3 NA objective was used. The microscope was controlled by Axiovision 4.8.1 software. For detailed analyses of border cell migration and to verify first-round hits, GAL4/UAS-RNAi crosses were independently set up, and whole ovaries from the adult progeny were fixed and stained for Singed, phalloidin, and DAPI as above. Manually dissociated ovaries were mounted on slides and analyzed as above using the same microscope.

### Calculation of Stat92E/DAPI intensity ratio

Border cell clusters stained with anti-Stat92E and DAPI were imaged with multiple optical *z*-sections. A maximum intensity projection image was generated using the Axiovision Extended Focus module. For overlapping nuclei, separate projection images were generated to visually isolate the nuclei. Individual border cell nuclei were first outlined in NIH ImageJ software. The mean DAPI and Stat92E intensities were measured using the “Measure” command in ImageJ. The ratio of Stat92E/DAPI for each nuclei was calculated by dividing the mean Stat92E intensity by the mean DAPI intensity.

### Graphs, statistics, and figures

Graphs and statistical analysis were performed in GraphPad Prism 4. The threshold for determining RNAi-induced migration phenotypes was calculated by applying the three-sigma rule on the negative control data (*c306-GAL4/+*; *UAS-mCD8:GFP/UAS-GFP dsRNA*). Briefly, the background migration defect was 2.63 ± 2.32% (mean ± SD; data from seven trials, n ≥ 50 egg chambers per trial). The threshold was calculated to be 9.59%, three standard deviation (SD) intervals from the mean. Statistical significance of RNAi knockdown by qRT-PCR was determined using the one-tailed unpaired *t*-test. In all other cases, the two-tailed unpaired *t*-test was used. Figures were assembled in Adobe Illustrator CS5. Minor image adjustments (brightness and/or contrast) were done in Axiovision 4.8.1 or Adobe Photoshop CS5. Gene ontology analyses were performed using PANTHER (http://www.pantherdb.org) (Thomas *et al.* 2003) or AmiGO (http://amigo.geneontology.org) (Ashburner *et al.* 2000).

## Results

### RNAi knockdown of PDZ domain-encoding genes in border cells

We first sought to identify all of the Drosophila genes that encode PDZ domain-containing proteins. We used a combination of the InterPro protein signatures (http://www.ebi.ac.uk/interpro/) and FlyBase (http://flybase.org/) databases to identify genes that have at least one PDZ domain. While the human genome encodes more than 250 PDZ domain proteins (Tonikian *et al.* 2008), Drosophila has 66 PDZ genes (Table S1) (Bilder 2001). Many of these genes have alternatively spliced isoforms, making the total number of PDZ domain proteins slightly higher (Sierralta and Mendoza 2004). We performed a gene ontology analysis to determine the types of proteins that these genes encode along with predicted functions (see *Materials and Methods*). Drosophila PDZ proteins are annotated predominantly to have protein binding, structural roles and regulation of enzyme activities (Figure S1). The relatively small number of PDZ domain genes in the Drosophila genome makes it a reasonable pool of candidates to test comprehensively for their role in border cell migration.

Knockdown of the multi-PDZ domain protein Baz, which regulates border cell migration, was used as a positive control (Figure 1 and Table S1) (Pinheiro and Montell 2004). We tested different GAL4 drivers to determine the best one for UAS-RNAi knockdown. A ubiquitous GAL4 driver, *tubulin*-GAL4, was lethal with *baz* RNAi (line v2914) and therefore was not used. We next tested a follicle cell driver, *T155*-GAL4, which is expressed early in the germarium in follicle cell precursors followed by expression in all follicle cells starting at stage 9 (Queenan *et al.* 1997; Liu and Montell 1999). We observed a high proportion of follicle cell defects when *baz* RNAi was driven by *T155*-GAL4 (Figure 1, B and C; 33%, n = 136 egg chambers). Regions of the follicle cell epithelium were thin (Figure 1B) and multilayered (Figure 1C). In addition, some follicle cells did not complete their posterior-directed retraction to cover the oocyte at stage 10B (Figure 1C). These results are consistent with the known role for Baz in follicle cell polarity (Cox *et al.* 2001; Huynh *et al.* 2001; Abdelilah-Seyfried *et al.* 2003) and indicates that the RNAi line efficiently knocked down *baz* function. However, we also observed a large proportion of degenerating egg chambers (33%, n = 136), which precluded us from scoring border cell migration.

We next tested two GAL4 drivers, *slbo*-GAL4 and *c306*-GAL4, which are expressed during border cell migration (Figure 1, D and E) (Murphy and Montell 1996; Rørth *et al.* 1998). We compared the expression patterns by crossing the GAL4 lines to UAS-mCD8:GFP. *slbo*-GAL4 begins to drive expression at high levels in the newly formed border cells at early stage 9 (Figure 1D). In contrast, *c306*-GAL4 turns on at earlier stages, beginning around stage 4/5 (Figure 1E). *c306*-GAL4 is expressed in a larger subset of follicle cells at the anterior end of the egg chamber that includes the presumptive border cells (Figure 1, A and E). Both GAL4 lines are also expressed in a subset of posterior follicle cells (Figure 1, D and E). We compared the border cell migration defects caused by knockdown of *baz* RNAi (line v2914) using *slbo*-GAL4 and *c306*-GAL4. This was scored as the percentage of border cells that migrated (complete) or did not migrate (incomplete) to the oocyte by stage 10 of oogenesis (Figure 1, A and F). We consistently observed stronger inhibition of migration with *baz* RNAi driven by *c306*-GAL4 (Figure 1F), possibly because the earlier follicle cell expression allowed time for efficient knockdown of gene function in the presumptive border cells. Therefore, we selected *c306*-GAL4 for this study (Figure 1G).

We obtained available transgenic UAS-RNAi lines that target each of the PDZ domain-encoding genes from VDRC (Figure 1G and Table S1). A few lines were acquired from two other collections (NIG-Fly and TRiP). Whenever possible, multiple transgenic RNAi lines that target each gene were obtained. RNAi lines can potentially produce off-target effects by non-specific knockdown of other genes (Perrimon *et al.* 2010). We excluded a few lines that were annotated to have a high number of potential off-target genes (more than 100), but in most cases we were able to test alternative lines. For example, *CG43955* had one line (v31686) with 196 predicted off-target genes including *taiman*, which is required for border cell migration (Bai *et al.* 2000); we tested the alternative line v103267, which does not have any predicted off-target genes. The only available *arc* RNAi line (v16826) has 408 predicted off-targets that include *taiman* and another border cell migration gene, *slow border cells* (*slbo*) (Montell *et al.* 1992), so it was not included in the PDZ gene survey. No lines were available for *Mhcl*. For the remaining 64 genes, 145 lines were tested for incomplete *vs.* complete border cell migration (Figure 1, A and G, and Table S1). More than 75% of the genes had multiple RNAi lines, either independent insertions of the same construct (*e.g. baz*) or independent constructs (*e.g. bbg*) (Table S1). We knocked down the genes using UAS-RNAi lines driven by *c306*-GAL4 (see *Materials and Methods*). *baz* RNAi was used as a positive control (Figure 1, A and F). RNAi against GFP was used as the negative control and did not significantly disrupt border cell migration (Figure 1F).

The percentage incomplete migration for all of the tested lines is reported in Table S1. The results were classified into two main groups, positive or negative hits. We calculated the minimum threshold for migration defects using our negative control data (see *Materials and Methods*); migration defects of 9% or fewer of the analyzed egg chambers were considered negative hits. RNAi lines that resulted in more than 9% of egg chambers with border cell migration defects were classified as positive hits. To confirm the first-round positive hits, we retested the strongest RNAi lines for most genes (see *Materials and Methods* and Figure 1G). Four genes that initially were categorized as positive hits in the first round did not repeat (Table S1). Moreover, upon retesting, some positive lines had stronger migration defects, whereas others were milder; this suggests slight inherent variability of knockdown efficiency. Nonetheless, most RNAi lines when retested exhibited similar strength of migration defects; the variation between trials was generally ≤ 10% (Table S1). Thirty-three genes fell into the negative hit category with the rest being positive hits (Table S1). For the 31 positive gene hits, RNAi knockdown caused migration defects ranging from 10 to 50% of the analyzed egg chambers; none of the lines completely blocked migration. Positive hits were further sorted based on the number of tested lines that had border cell migration defects (Table S1). Genes with all or multiple RNAi lines producing migration defects were designated “high confidence” multiple hits (Table 1) (Booker *et al.* 2011). A total of 14 positive genes compose the multiple hit category (Table 1) and 17 genes are single hits (Table S1). The positive hit genes have a range of predicted functions, although genes with known or predicted roles in epithelial polarity or cytoskeletal regulation together account for more than half of the hits (Table 1 and Figure S1C).

**Table 1:** High-confidence PDZ domain-encoding genes in border cell migration identified by RNAi knockdown

Gene	Putative Vertebrate Homolog*^a^*	Other Domains Present*^b^*	No. of Hits / Total Lines*^c^*	Known Role in Cell Migration*^d^*
bazooka	PARD3 (PAR3)	Oligomerization domain	2 / 2	Pinheiro and Montell (2004)*^e^*
				Nakayama *et al.* (2008)*^f^*
big bang	PDZD2	None	2 / 3	—
CASK ortholog	CASK	Guanylate kinase domain	2 / 3	—
		L27 domain		
		Protein kinase, catalytic domain, inactive		
		SH3 domain		
CG5921	Harmonin / USH1C	None	2 / 2	—
CG6498	MAST2	Domain of unknown function	3 / 3	—
		Protein kinase, catalytic domain		
CG6509	DLG5	Guanylate kinase domain	2 / 3	Smolen *et al.* (2010)*^f^*
		Src homology 3 domain		
Gef26	RAPGEF2 / PDZ-GEF1	Cyclic nucleotide-binding domain	3 / 3	Huelsmann *et al.* (2006)*^e^*
		Guanine-nucleotide dissociation stimulator (RasGEF)		
		Ras association domain		
		Ras-like guanine nucleotide exchange factor, N-terminal		
Lap1	LRRC7 / ERBB2IP	Leucine-rich repeats	2 / 2	—
LIMK1	LIMK1	LIM zinc-binding domain	2 / 3	Zhang *et al.* (2011)*^e^*
		Protein kinase, catalytic domain		Nishita *et al.* (2005)*^f^*
par-6	PARD6 (PAR6)	PB1 domain	3 / 3	Pinheiro and Montell (2004)*^f^*
PatJ	INADL / MPDZ	L27 domain	2 / 3	Shin *et al.* (2007)*^f^*
Rim	RIMS2	C2 domain	2 / 3	—
stardust	MPP5 (PALS1)	Guanylate kinase domain	2 / 3	—
		L27 domain		
		Src homology 3 domain		
veli	LIN7A / LIN7B / LIN7C	L27 domain	2 / 2	—

a Putative homologs were found using NCBI Homologene and UniProt.

b Protein domains were identified using NCBI Conserved Domains Database and Interpro.

c Number of RNAi lines that resulted in a migration defect out of all lines tested.

d Cited references describe the Drosophila gene or its homologs.

e Pertains to studies in Drosophila.

f Pertains to the mammalian homolog.

### Validation of candidates

**Figure 2:**
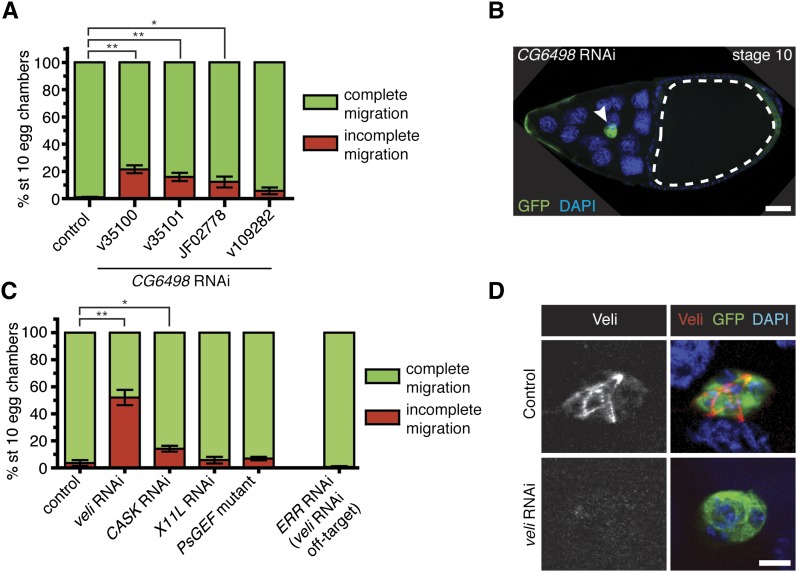
Confirmation of positive and negative hit genes. (A and C) Quantification of border cell migration at stage 10, shown as the percentage of border cells with complete (green) or incomplete (red) migration in egg chambers expressing RNAi to GFP (control) or the indicated RNAi transgenes driven by *c306*-GAL4. Error bars represent SEM; n ≥ 50 egg chambers in each of at least three trials (*P < 0.05; **P < 0.01; two-tailed unpaired *t*-test). (A) Knockdown of *CG6498* using multiple transgenes disrupts border cell migration. (B) Representative example of an egg chamber with a border cell migration defect caused by *CG6498* RNAi. Genotype is *c306-GAL4/*+; *UAS-mCD8:GFP/UAS-CG6498 RNAi v35100*. Border cells (green; arrowhead) stopped a little more than halfway to the oocyte (outlined). DAPI marks nuclei. Scale bar is 20 µm. (C) Border cell migration defects by RNAi knockdown of *veli* (v43094) and *CASK* (v34185). Normal border cell migration with RNAi knockdown of *X11L* (v28652) and in a *PsGEF* mutant (*PsGEF^Δ55^/PsGEF*^Δ21^). RNAi for *ERR* (line v108349), the predicted off-target gene for *veli* RNAi line v43094, did not disrupt border cell migration. (D) Border cells stained with an antibody to Veli. Control border cells (*c306-GAL4/*+; *UAS-mCD8:GFP/*+) had detectable Veli (red), which was strongly reduced in *veli* RNAi border cells (*c306-GAL4/*+; *UAS-mCD8:GFP/UAS-veli RNAi v43094*). GFP (green) shows GAL4 expression and DAPI (blue) labels nuclei. Scale bar is 10 µm.

To verify results from the systematic RNAi knockdown of PDZ genes, we performed additional tests for a subset of both positive and negative genes. We first performed a detailed analysis of the migration defects for selected positive first-round genes (see *Materials and Methods*). *CG6498* has high homology to human *microtubule-associated serine-threonine kinase 2* (*MAST2*) (NCBI Homologene; http://www.ncbi.nlm.nih.gov/homologene/); both genes encode proteins with a central serine-threonine kinase domain and a single PDZ domain. Three *CG6498* RNAi lines (two different constructs) significantly disrupted migration (Figure 2A; http://flybase.org/reports/FBgn0036511.html). One independent construct, line v109282, did not cause migration defects, possibly due to inefficient knockdown (Figure 2A). In the strongest line, v35100, 21% of the egg chambers had incomplete migration (Figure 2A). Most *CG6498* RNAi border cells with a migration defect stopped midway to the oocyte (Figure 2B). Moreover, knockdown of *CG6498* driven by the other border cell GAL4, *slbo*-GAL4, also disrupted border cell migration (Figure S2). We analyzed in more detail the migration defects caused by RNAi lines for two additional multi-hit positive genes, *veli* and *CASK* (Figure 2C). Closer examination of RNAi knockdown for both genes revealed migration defects similar to those observed in the first-round analysis (Figure 2C and Table S1). We confirmed that Veli was expressed in border cells using an antibody against Veli protein (Figure 2D). The strongest *veli* RNAi line (v43094) has a predicted off-target match to *estrogen receptor related* (*ERR*). However, RNAi for *ERR* did not disrupt border cell migration (Figure 2C). Moreover, *veli* RNAi (v43094) efficiently downregulated Veli levels in border cells (Figure 2D).

We next confirmed that two genes in the negative hit category did not disrupt border cell migration. *X11L* RNAi-induced phenotypes were close to the background cutoff migration defects observed in the first-round tests (Table S1). Upon detailed retesting, we verified that knockdown of *X11L* did not affect border cell migration (Figure 2C). We also obtained two small deletion mutant alleles of the putative Rac guanine-nucleotide exchange factor (GEF) *Protostome-specific GEF* (*PsGEF*); these alleles are transcript null, viable, and fertile (Higuchi *et al.* 2009). In agreement with the RNAi results, egg chambers *trans*-heterozygous for *PsGEF^Δ55^/PsGEF^Δ21^* were morphologically normal and did not disrupt border cell migration (Figure 2C).

Although most RNAi lines are expected to produce knockdown of the targeted genes, this has not been tested formally for most individual lines. Therefore, we performed qRT-PCR to ascertain the *in vivo* knockdown efficiency for selected RNAi lines. We analyzed 18 lines, which target 4 positive genes and 14 negative genes; this encompasses ∼25% of the PDZ genes (Table 2). The tested lines were from multiple collections: the first-generation “GD” and second-generation “KK” long double-stranded hairpin RNA (dsRNA) libraries from VDRC; and the long dsRNA (Valium 1 and 10) and shRNA (Valium 20) libraries from the TRiP collection (http://flybase.org/reports/FBrf0208510.html) (Dietzl *et al.* 2007; Ni *et al.* 2009; 2011). To determine whether these genes were expressed during oogenesis, qRT-PCR was used to measure the relative expression levels in wild-type ovarian extracts (Table 2). Most genes were expressed at low or low-to-moderate levels, with the exception of *CG43955*, which was not expressed. Next, we crossed the 18 RNAi lines to *heat shock* (*hs*)-GAL4 and subjected adult flies to heat shock to induce RNAi transgene expression (see *Materials and Methods*). RNA isolated from whole female flies (ovaries removed) was used to analyze relative levels of transcript in RNAi knockdown flies *vs.* a non-targeting control (RNAi to GFP). qRT-PCR performed on the resulting cDNA showed that 14 out of 18 RNAi lines achieved statistically significant knockdown of transcripts (Table 2 and Figure S3). Knockdown ranged from mild (21% knockdown by *PICK1* RNAi) to strong (90% knockdown by *Grip* RNAi), with most lines producing more than 40% knockdown. Finally, we compared lines we tested to those identified in two genome-wide *in vivo* RNAi screens performed to identify genes that regulate Notch signaling or muscle morphogenesis (Mummery-Widmer *et al.* 2009; Schnorrer *et al.* 2010). These genome-wide screens found specific phenotypes or lethality with 12 positive hit lines and 9 negative hit lines from our analysis (Table S1). Together, these data confirm specificity for a number of RNAi lines. Moreover, this suggests that the majority of lines tested in this study are expected to reduce relevant transcript levels.

**Table 2:** Expression levels and RNAi knockdown efficiency as measured by quantitative RT-PCR

Gene	Expression Level in the Ovary (C_T_ value ± SD)*^a^*^,^*^b^*	RNAi Line*^c^*	Percentage Knockdown in Whole Flies*^d^*
Positive candidates			
baz	25.7 ± 0.350 (L–M)	v2914	41***
bbg	27.4 ± 0.435 (L–M)	v15975	89**
CG6498	26.0 ± 0.640 (L–M)	v35100	53*
CG6509	26.3 ± 0.868 (L–M)	v22496	77**
Negative candidates			
CG43707	34.5 ± 0.885 (L)	v25846	40 (ns)
CG43955	40.1 ± 1.51 (NE)	v103267	78**
CG9588	24.3 ± 0.318 (M–H)	HM05013	82***
cnk	25.5 ± 0.451 (L–M)	HMS00238	67***
dlg1	25.4 ± 0.575 (L–M)	HMS01521	52**
dysc	28.6 ± 0.237 (L–M)	v23278	32*
Grip	28.4 ± 0.671 (L–M)	v103551	90***
PICK1	30.4 ± 0.252 (L)	JF01199	21**
RHOGAP100F	31.9 ± 0.874 (L)	HMS00740	14 (ns)
scrib	25.8 ± 0.512 (L–M)	v105412	50**
sif	31.2 ± 0.0700 (L)	v106832	16 (ns)
Syn1	31.2 ± 0.500 (L)	JF02654	32 (ns)
vari	25.7 ± 0.0985 (L–M)	HM05087	72***
X11L	26.0 ± 0.463 (L–M)	v28652	55**

a Mean C_T_ value and SD are calculated from three independent qPCR experiments. Expression summary: C_T_ < 20, very high (VH); C_T_ = 20–25, moderate to high (M–H); C_T_ = 25–30, low to moderate (L–M); C_T_ = 30–35, low (L); C_T_ = 35–40, none to low (N–L); C_T_ > 40, not expressed (NE).

b For reference, the endogenous expression levels (mean C_T_ value ± SD) of *rp49* and *tub84b* were 17.7 ± 0.112 and 19.1 ± 0.16, respectively.

c RNAi lines are from the Vienna Drosophila RNAi Center (prefixed with v) and from the Harvard Transgenic RNAi Project (prefixed with HM, HMS, or JF).

d Extent of reduction of target gene expression compared with gfp dsRNA control, calculated using the ΔΔC_T_ method with *rp49* expression as reference. The one-tailed unpaired *t*-test was used to test for significance (ns, *P* > 0.05; ^*^P = 0.01–0.05; ^**^P = 0.001–0.01; ^***^P < 0.001).

### Investigation of two genes, *bbg* and *CG6509*, reveals distinct functions in border cells

While the initial analysis of PDZ domain-encoding genes by RNAi knockdown focused on whether border cells completed their migration by the appropriate stage, we wanted to further determine specific function(s) of identified genes in border cells. Earlier studies established that Baz and its partner Par-6 regulate polarity of border cells during detachment from the follicle cell epithelium and subsequent migration (Pinheiro and Montell 2004; McDonald *et al.* 2008). None of the other high-confidence positive hits from this study have been analyzed previously in border cells, although a few have been found to regulate the migration of other cell types in Drosophila and/or in mammals (Table 1). Two genes, *big bang* (*bbg*) and *CG6509*, were chosen for further tests because they encode different classes of PDZ domain-containing proteins.

**Figure 3:**
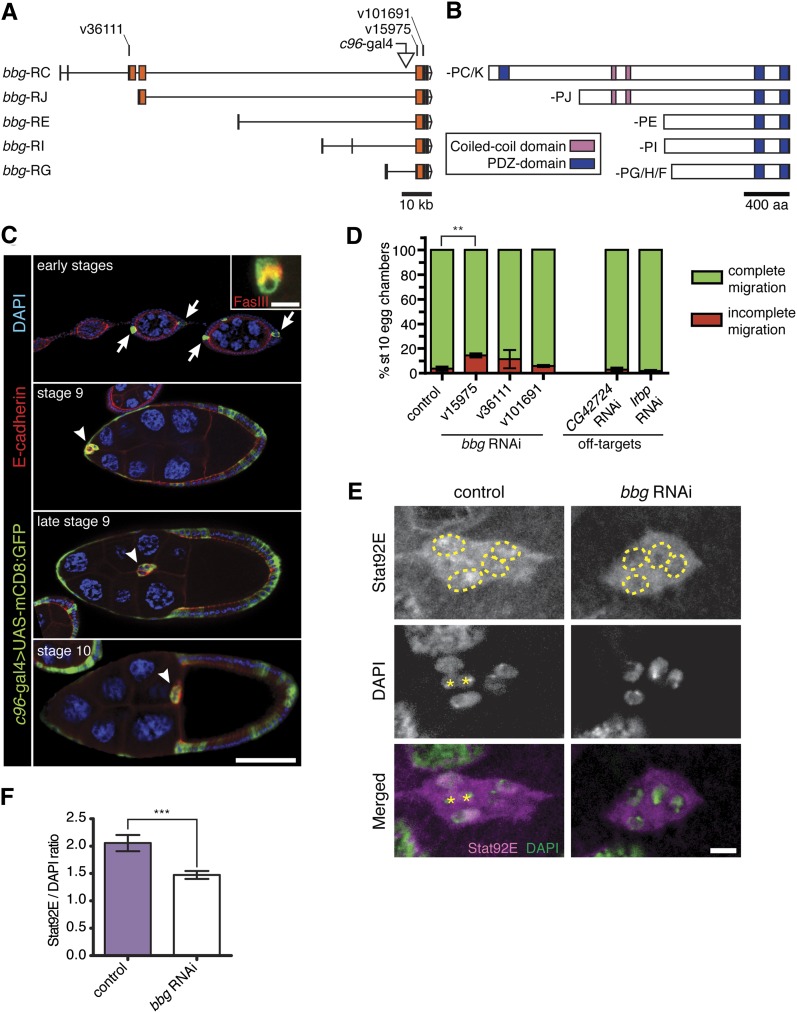
The multi-PDZ domain protein Big bang regulates nuclear STAT levels in border cells. (A) Schematic diagram of five *bbg* predicted transcripts (adapted from FlyBase); coding exons are in orange. RNAi target sequences and *c96*-GAL4 insertion site are indicated. RNAi lines v15975 and v101691 target sequences common to all isoforms. RNAi line v36111 is specific to RC and RK (not shown; differs from RC only in a non-coding exon; see FlyBase) transcripts. (B) Schematic diagram of the eight Bbg protein isoforms, which have either two or three PDZ domains. (C) Egg chambers showing *c96*-GAL4 expression pattern visualized by UAS-mCD8:GFP (green) at the indicated stages. Egg chambers were co-stained for E-cadherin (red) to mark cell membranes and DAPI (blue) to mark nuclei. Scale bar is 50 µm. (Top panel) *c96*-GAL4 expression in anterior and posterior polar cells (arrows) at early stages. Inset shows *c96*-GAL4–positive polar cells (green) co-stained for FasIII (red; scale bar, 5 µm). (Bottom panels) *c96*-GAL4-driven GFP in border cells (arrowheads) and surrounding follicle cell epithelium during stages 9 and 10. (D) Quantification of border cell migration at stage 10, shown as the percentage of border cells with complete (green) or incomplete (red) migration in egg chambers expressing RNAi to GFP (control) or the indicated RNAi transgenes driven by *c306*-GAL4. Knockdown of *bbg* with RNAi line v15975 disrupted border cell migration. *bbg* RNAi line v36111 had variable effects, and line v101691 did not disrupt migration. RNAi to the predicted off-target genes, *CG42724* (line v30629) and *Irbp* (line JF03273), did not disrupt migration. Error bars represent SEM; n ≥ 50 egg chambers in each of at least three trials (**P = 0.0071; two-tailed unpaired *t*-test). (E) Reduction of Stat92E levels in border cell nuclei when *bbg* is knocked down. Stage 9 border cells stained for Stat92E (magenta) and DAPI (green). Stat92E is expressed at higher levels in control border cell nuclei (yellow outline) compared with cytoplasm (*c306-GAL4/*+; *UAS-mCD8:GFP/*+). Stat92E is expressed at low levels in *bbg* RNAi (*c306-GAL4/*+; *UAS-mCD8:GFP/UAS-bbg RNAi v15975*) border cell nuclei (outlined). Polar cells (asterisks) were excluded from analyses. Scale bar is 5 μm. (F) Quantification of the fluorescence intensity ratio of STAT nuclear staining to DAPI staining for control (n = 59) or *bbg* RNAi (n = 88) border cells; genotypes as in (E). At least 16 individual clusters were analyzed. Error bars represent SEM (***P < 0.001; two-tailed unpaired *t*-test).

*bbg* encodes a large multi-PDZ domain protein expressed at various stages of development (Kim *et al.* 2006). Little is known about the function of *bbg* in development, except that mutants are mildly bang sensitive (Kim *et al.* 2006). The *bbg* gene locus spans over 120 kb and encodes multiple transcripts (5 of the 8 are shown; Figure 3A) (Kim *et al.* 2006). Bbg protein isoforms are differentiated by the total number of PDZ domains present (three in Bbg-PC/-PK, two in the other isoforms), by variations in the length of the N-terminal region, and the presence of two predicted coiled-coil domains (Figure 3B) (Kim *et al.* 2006).

*bbg* is expressed in early oogenesis as well as in discrete patterns in the embryo and larval discs (Gustafson and Boulianne 1996; Kim *et al.* 2006). We used qRT-PCR to verify that *bbg* was expressed in ovaries (Table 2). However, its expression during later stages of oogenesis has not been described. *bbg* was previously identified as the insertion site for the *c96*-GAL4 enhancer trap line, which has been shown to reliably report the *bbg* expression pattern (Kim *et al.* 2006). We analyzed the expression of *bbg* by crossing *c96*-GAL4 to UAS-mCD8:GFP (Figure 3C and Figure S4A). *c96*-GAL4 was restricted to a few follicle cells at the very anterior and posterior ends of the egg chamber at early stages (Figure 3C), in agreement with *bbg* transcript and protein (Kim *et al.* 2006). Staining with Fasciclin III (FasIII), which marks the membrane between the pair of polar cells, confirmed that these cells are the anterior and posterior polar cells (Figure 3C). The polar cells later recruit surrounding follicle cells to become border cells at late stage 8 (Silver and Montell 2001; Xi *et al.* 2003). Starting at stage 8, *c96*-GAL4-driven GFP expanded to the majority of follicle cells, including border cells (Figure 3C and Figure S4A).

We next confirmed that *bbg* RNAi-mediated knockdown disrupted border cell migration (Figure 3D). We retested three transgenic RNAi lines that target non-overlapping regions of *bbg* (Figure 3A). Line v15975 resulted in the strongest migration defects (14% of stage 10 egg chambers), whereas line v36111 had milder and more variable migration defects (Figure 3D). Upon retesting, the third RNAi line against *bbg* (v101691) did not reliably disrupt migration. Knockdown of *bbg* using *slbo*-GAL4 mildly disrupted border cell migration (Figure S2). The first two RNAi lines against *bbg* each have a predicted off-target gene. However, *Irbp* RNAi (off-target for v15975) and *CG42724* RNAi (off-target for v36111) did not induce migration defects (Figure 3D). Moreover, *bbg* RNAi line v15975 significantly knocked down *bbg* levels *in vivo* (Table 2 and Figure S3).

**Figure 4:**
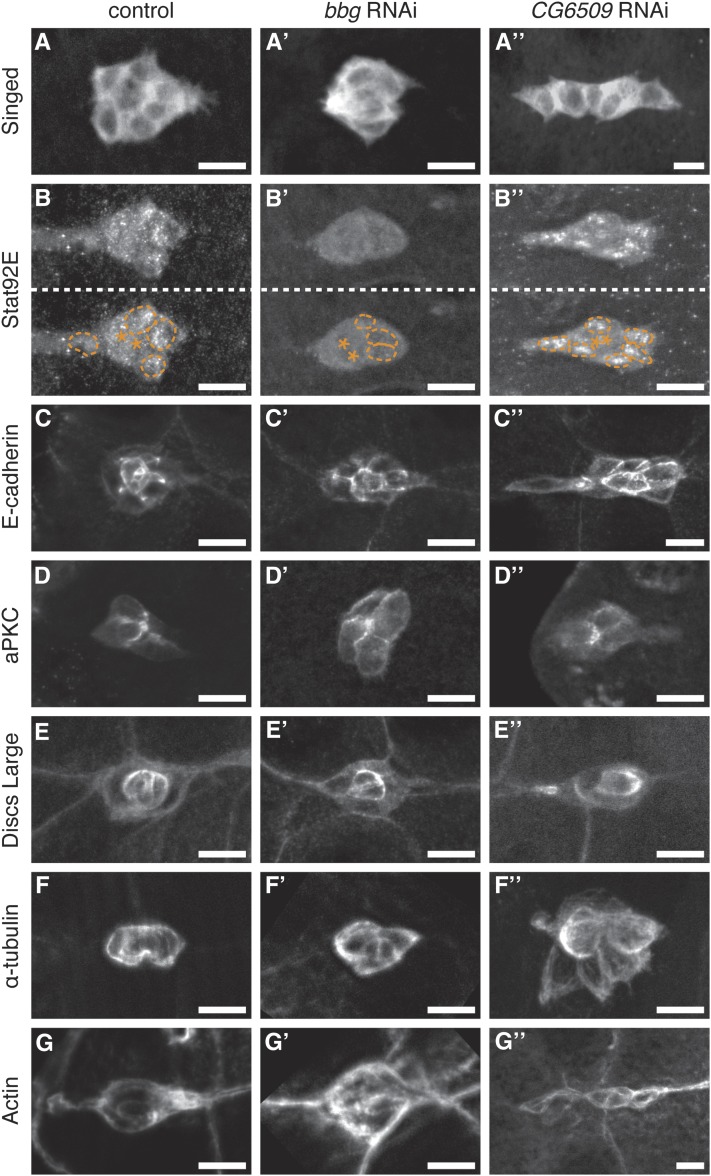
Markers of cell fate, cell adhesion, polarity, and cytoskeleton in *bbg* RNAi and *CG6509* RNAi border cells. Representative immunofluorescent images of stage 9 control (*c306-GAL4/*+; *UAS-mCD8:GFP/*+), *bbg* RNAi (*c306-GAL4/*+; *UAS-mCD8:GFP/UAS-bbg RNAi v15975*), and *CG6509* RNAi (*c306-GAL4/*+; *UAS-mCD8:GFP/UAS-CG6509 RNAi v22496*) border cells. (A and B) Border cells stained for antibodies to the cell fate markers Singed (A) and Stat92E (B). (A) Singed is enriched in the cytoplasm. (B) Stat92E is enriched in border cell nuclei compared with the cytoplasm. The same cluster is presented with and without border cell nuclei outlined with a dotted line (taken from DAPI staining of nuclei, not shown). Polar cells are marked with an asterisk (*). (C) Border cells stained for the cell adhesion protein E-cadherin, which is high in central polar cells and at the membrane interface between border cells. (D and E) Border cells stained for the cell polarity proteins aPKC (D) and Dlg1 (E). (D) aPKC is an apical cell marker and localizes between border cells; an apical view is shown. (E) Dlg1 is a basolateral cell marker that is enriched in the central polar cells and at lower levels at border cell membranes. (F and G) Border cells stained for the cytoskeletal markers α-tubulin to mark microtubules (F) and phalloidin to label F-actin (G). N ≥ 10 border cell clusters assayed for each genotype. Scale bar is 10 µm.

To address whether Bbg regulates a specific aspect of border cell migration, we analyzed the levels and localization of several border cell-enriched proteins (Figure 4). We first analyzed a marker of cell identity, the fascin homolog Singed (Sn). Border cells in which *bbg* was knocked down by the strongest RNAi line (v15975) had normal levels and localization of Sn compared with control border cells (Figure 4A). The cell adhesion protein E-cadherin and the membrane-associated polarity proteins atypical protein kinase C (aPKC) and discs large 1 (Dlg1) were all localized correctly in *bbg* RNAi border cells (Figure 4, C–E). Moreover, *bbg* RNAi did not disrupt F-actin or α-tubulin, indicating no obvious cytoskeletal defects (Figure 4, F and G). Thus, most aspects of border cell differentiation and membrane localization were unchanged when *bbg* levels were reduced. In contrast, we observed altered Stat92E subcellular localization when *bbg* was knocked down (Figures 3E and 4B).

JAK/STAT signaling specifies border cell fate, recruits border cells to form a cluster, and promotes their motility (Silver and Montell 2001; Beccari *et al.* 2002; Silver *et al.* 2005). Nuclear STAT localization reflects high levels of JAK/STAT signal activation (Vinkemeier 2004). Stat92E (the Drosophila STAT homolog) becomes enriched in border cell nuclei as they are specified in the epithelium and is maintained throughout their migration (Silver *et al.* 2005). Control migrating border cells have visibly higher nuclear Stat92E compared with the cytoplasm (Figure 3E). However, *bbg* RNAi reduced the levels of nuclear Stat92E in most border cells (Figure 3E). To quantitate this effect, we measured the ratio of nuclear Stat92E to DAPI staining in stage 9 migrating border cells (see *Materials and Methods*). The ratio of nuclear STAT to DAPI signal was reduced from ∼2.0 in control border cells to ∼1.5 in *bbg* RNAi border cells (Figure 3F). Nonetheless, high nuclear Stat92E was observed in premigratory *bbg* RNAi border cells (Figure S4B). This result suggests that nuclear Stat92E was initially normal in *bbg* RNAi border cells but that it was not maintained adequately after border cells began to migrate. *bbg* RNAi border cell clusters contain a similar number of cells compared with control clusters (Figure S4C). Thus, Bbg functions after border cells are specified and recruited into the cluster to maintain optimal STAT levels during border cell migration.

**Figure 5:**
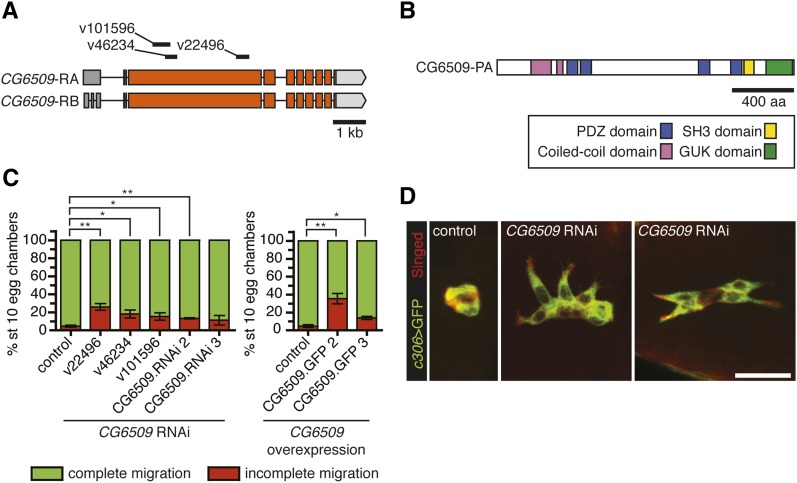
The MAGUK family member CG6509 regulates border cell cluster morphology. (A) Schematic of the *CG6509* transcripts, which differ only in the 5′ non-coding exons (adapted from FlyBase); coding exons in orange. RNAi target sequences are indicated. (B) Schematic of CG6509 protein showing the conserved domains. (C) Quantification of border cell migration at stage 10, shown as the percentage of border cells with complete (green) or incomplete (red) migration in egg chambers expressing multiple RNAi lines or overexpression of full-length UAS-CG6509 (different insertions of same transgene) driven by *c306*-GAL4. Error bars represent SEM; n ≥ 50 egg chambers in each of at least three trials (*P < 0.05; **P < 0.01; two-tailed unpaired *t*-test). (D) Stage 9 border cells stained for GFP (green) and Singed (red) to reveal border cell cluster morphology. Representative example of a control (*c306-GAL4/*+; *UAS-mCD8:GFP/*+) border cell cluster. Two examples of *CG6509* RNAi (*c306-GAL4/*+; *UAS-mCD8:GFP/UAS-CG6509 RNAi v22496*) border cells in which the cluster is partially dissociated (middle panel) or elongated (right panel). Scale bar is 20 μm.

The second gene we analyzed in more detail, *CG6509*, encodes a member of the membrane-associated guanylate kinase (MAGUK) proteins (Figure 5, A and B). The domain architecture of CG6509, a combination of PDZ, SH3, and guanylate kinase (GUK) homology domains, is characteristic of members of the MAGUK family of scaffolding proteins (Oliva *et al.* 2012). Four other MAGUK-encoding genes were identified as positive hits: the multi-hit genes *CASK ortholog* (*CASK*) and *stardust* (*sdt*) and the single-hit genes *menage a trois* (*metro*) and *polychaetoid* (*pyd*). We verified that knockdown of *CG6509* with multiple RNAi lines disrupted border cell migration (Figure 5C). Although the RNAi line v22496 initially fell below the migration defect cutoff, upon retesting, it inhibited border cell migration in 26% of stage 10 egg chambers (Figure 5C and Table S1). Moreover, line v22496 produced significant knockdown of *CG6509* transcript levels (Table 2 and Figure S3). Knockdown of *CG6509* using the border cell-specific *slbo*-GAL4 mildly delayed migration, confirming a requirement in border cells (Figure S2). Three *CG6509* RNAi lines (v22496, v46234, and v101596) do not have predicted off-target genes, further indicating that the phenotypes are specific to *CG6509* knockdown.

Analysis of border cell markers in *CG6509* RNAi border cells, similar to that performed for *bbg* (see above), did not reveal obvious changes compared with control (Figure 4). Nonetheless, RNAi knockdown of *CG6509* markedly affected the morphology of border cell clusters (Figures 4 and 5D). Control clusters are generally round and fairly compact (96%; n = 21). In contrast, 44% of *CG6509* RNAi border cell clusters (n = 36) no longer had a compact shape and, instead, were dissociated or elongated (Figure 5D). Finally, overexpression of *CG6509* in border cells also disrupted their migration; the strongest UAS-*CG6509* line disrupted migration in 35% of stage 10 egg chambers (Figure 5C). These data together indicate that having proper levels of CG6509 is important for normal border cell migration and cohesion of the cluster.

## Discussion

### RNAi knockdown of specific classes of genes identify regulators of border cell migration

The advantage of using RNAi to test a selected class of genes, like the one performed here, is the rapid identification of genes involved in a particular process such as border cell migration. The less labor-intensive nature of this approach ensures that even those genes whose knockdown results in incompletely penetrant phenotypes are detected. Recently, this method was used to identify Evi5 as a new GTPase-activating protein for Rab11 in border cell migration (Laflamme *et al.* 2012). Moreover, targeted RNAi knockdown of microtubule-associated proteins demonstrated a requirement for the Lis-1 complex in border cells (Yang *et al.* 2012). In this study, we chose to systematically target 64 genes that encode PDZ domain-containing proteins because of their known functions in processes critical for cell migration, such as cell polarity, adhesion, and signaling. The majority of these PDZ genes have not been examined for functions in cell migration in any organism. The 14 genes designated as high-confidence hits likely represent new members of protein complexes required for border cell migration. Importantly, several positive genes, the multi-hit genes *baz* and *par-6* and the single-hit gene *dishevelled* (*dsh*), were previously identified as regulators of border cell migration (Pinheiro and Montell 2004; Bastock and Strutt 2007). Most genes in the positive class, which includes 17 additional genes with one phenotypic RNAi line, have direct or putative mammalian homologs. The results of this study thus provide a list of PDZ genes whose roles in cell migration and motility are predicted to be conserved.

Knockdown of positive hit PDZ genes resulted in mild to moderate migration defects, with most border cells able to detach from the epithelium and migrate partway to the oocyte. These observations suggest that RNAi for these genes resulted in partially penetrant phenotypes, either due to partial knockdown of gene function or because the gene is not completely essential for full border cell motility. Incomplete knockdown could occur if the RNAi transgene is not expressed at the right time or at strong enough levels (Perrimon *et al.* 2010; Booker *et al.* 2011). To overcome this potential problem, the *c306*-GAL4 driver was used because it is expressed early in follicle cells and maintained in the migrating border cells. Whenever possible, multiple independent insertion lines and/or constructs were tested to minimize the potential issue of inefficient RNAi constructs. Multiple independent hits increase the likelihood that the migration defects caused by RNAi are specific. Our assessment of RNAi efficiency by qRT-PCR confirms that 75% of the tested RNAi lines effectively knocked down the relevant targeted transcript. While knockdown efficiency ranged from 20 to 90%, most RNAi lines decreased transcript levels by 40% or more. Furthermore, our results indicate that, at least for *baz*, partial knockdown (∼40% reduction of transcripts) significantly disrupted border cell migration.

The relatively mild nature of the phenotypes alternatively suggests that these genes have support or partially redundant roles in border cell migration. A striking example of this is the two receptor tyrosine kinases, the epidermal growth factor receptor (EGFR) and the PDGF/VEGF receptor related (PVR), that guide border cells to the oocyte in response to secreted growth factors (Duchek and Rørth 2001; McDonald *et al.* 2006). Loss of either receptor alone has modest effects, but simultaneous loss of both receptors severely inhibits posterior-directed migration (Duchek and Rørth 2001; Duchek *et al.* 2001; McDonald *et al.* 2003). This contrasts with other genes, such as *slbo*, that are required for early border cell fate and whose loss completely inhibits migration (Montell *et al.* 1992). It remains to be seen whether the genes identified in this study play partially redundant roles. Many of the genes identified lack classical mutant alleles, and therefore, RNAi is the most direct method to assess their functions at present. Once loss-of-function alleles are tested and/or created, the mutant results can be compared with the RNAi knockdown results. In the future, it will also be important to identify the cellular and membrane-associated proteins to which these PDZ domain proteins bind. The results from this study accordingly present a collection of candidates to search for PDZ-interacting substrates in border cells and other migratory cells.

### Epithelial polarity and cytoskeletal-associated genes are highly represented hits

A key group of genes identified here are those involved in epithelial cell polarity. Significantly, these and other epithelial polarity proteins are required for mammalian cell motility and have been implicated in tumor invasion and metastasis (Etienne-Manneville 2008; Hidalgo-Carcedo *et al.* 2011; Subbaiah *et al.* 2011; Martin-Belmonte and Perez-Moreno 2012). Border cells retain many epithelial characteristics during migration, including polarized localization of Par-6 and Baz and upregulation of E-cadherin (Niewiadomska *et al.* 1999; Pinheiro and Montell 2004). Nine of the positive-hit genes (*baz*, *CASK*, *dsh*, *Lap1*, *par-6*, *Patj*, *pyd*, *sdt*, and *veli*) regulate apical-basal polarity to establish distinct membrane domains (Ashburner *et al.* 2000; Guillemot *et al.* 2008; Martin-Belmonte and Perez-Moreno 2012). This raises the possibility that these polarity proteins regulate the localization of junctional proteins in border cells to organize and promote migration, similar to the known functions of *baz* and *par-6* (Pinheiro and Montell 2004; Llense and Martín-Blanco 2008). Many of the proteins in the polarity group form known multi-protein complexes. Par-6 and Baz form a complex with the serine-threonine kinase aPKC in some contexts, and Sdt, Patj, and Veli form another complex with the transmembrane protein Crumbs (Martin-Belmonte and Perez-Moreno 2012). The identification of multiple members of these complexes in this study indicates that specific complexes function in border cell migration and confirms the sensitivity of this approach.

Notably, most of the polarity genes with phenotypes encode proteins that are associated with apical junctions of epithelial cells, for example, the Baz and Crumbs complexes, rather than basolateral junctions (Laprise and Tepass 2011; Martin-Belmonte and Perez-Moreno 2012). Two basolateral polarity complex proteins that contain PDZ domains, Dlg1 and Scribbled (Scrib), regulate mammalian epithelial cell migration (Dow and Humbert 2007). Moreover, Dlg1 is highly expressed in follicle cells and border cells (Szafranski and Goode 2004). Surprisingly, *dlg1* or *scrib* RNAi did not disrupt border cell migration even though their transcript levels were significantly knocked down. These proteins suppress cell invasion in ovarian follicle cells (Goode and Perrimon 1997; Szafranski and Goode 2007) and in a model of tumor invasion (Pagliarini and Xu 2003), thus they may have a different role in border cells. Indeed, loss of *dlg1* depolarizes the follicle cell epithelia, induces uncontrolled invasion, and may even stimulate border cell motility (Goode and Perrimon 1997; Szafranski and Goode 2004). Therefore, the activity of Dlg1, and possibly Scrib, likely needs to be downregulated in border cells to allow their detachment and migration. We previously found that the basolateral protein Par-1 is required for detachment of border cells from the follicle cell epithelium and their subsequent motility (McDonald *et al.* 2008). Therefore, border cells may use a different set of basolateral polarity proteins for migration compared with other types of epithelial cells.

The other major group of genes identified in this study encodes proteins with known or predicted roles in cytoskeletal regulation. This is consistent with established roles for the actin cytoskeleton and microtubules in migrating cells (Kaverina and Straube 2011; Ridley 2011). Like most migrating cells, border cells normally extend and retract actin-rich cellular protrusions that provide traction for migration and help them sense directional guidance cues (Murphy and Montell 1996; Fulga and Rørth 2002; Prasad and Montell 2007). Several microarray screens identified an enrichment of cytoskeletal-associated proteins in border cells compared with non-migratory cells (Borghese *et al.* 2006; Wang *et al.* 2006). Moreover, regulators of actin and microtubules promote the formation of dynamic border cell protrusions (Zhang *et al.* 2011; Kim *et al.* 2011; Yang *et al.* 2012). The cytoskeletal regulator *Lim Kinase 1* (*LIMK1*), which encodes a serine-threonine kinase with two LIM domains in addition to a single PDZ domain, was a multi-hit gene identified by our study. LIMK1 functions downstream of the Rac GTPase to regulate actin dynamics through the actin-regulatory protein cofilin (Bernard 2007). Moreover, LIMK1 mildly rescues the border cell migration defects caused by inactivation of Rac (Zhang *et al.* 2011). Our results demonstrate that LIMK1 itself is required for border cell migration. However, more work is needed to determine the extent to which LIMK1 functions primarily through Rac in border cells, as has been proposed (Zhang *et al.* 2011), or has any additional functions. Two Rac-GEFs, *myoblast city* and *elmo* (*Ced-12*), are required for border cell migration (Bianco *et al.* 2007; Geisbrecht *et al.* 2008). In contrast, we found that another putative Drosophila Rac-GEF (Higuchi *et al.* 2009), *PsGEF*, is not required for border cell migration. These results highlight the complex roles of Rac-effector proteins in specific cell and tissue contexts. Further investigation of the cytoskeletal-associated genes identified in this study is anticipated to provide new insights into the regulation of border cell motility.

### Roles of Bbg and CG6509 in cell migration

Although the targeted RNAi survey of PDZ gene function was designed to focus only on the extent of border cell migration, studies with *bbg* and *CG6509* revealed genes that regulate distinct features of border cells. Our results indicate that Bbg regulates levels of active Stat92E within migrating border cells. Activation of the JAK/STAT pathway in the follicle cells surrounding the polar cells is the first step in the specification of border cell fate and recruitment of cells to form the border cell cluster (Silver and Montell 2001). Subsequently, JAK/STAT signaling is actively maintained during migration (Silver *et al.* 2005). It was unclear from previous studies what mechanisms control nuclear Stat92E levels in border cells, although both active transport of Upd ligand mRNA and endocytosis appear to be important (Silver *et al.* 2005; Van de Bor *et al.* 2011). Despite the relatively mild migration defect caused by *bbg* knockdown, partially migrated *bbg* RNAi border cells exhibited reduced nuclear Stat92E and presumably reduced JAK/STAT activation. Moreover, STAT levels were unaffected in border cells prior to migration. Thus, Bbg is a new regulator of JAK/STAT signaling that upregulates and/or maintains nuclear Stat92E levels in migrating border cells. As the protein interaction partners of Bbg have yet to be identified, the mechanism for Bbg-mediated regulation of STAT activity remains to be elucidated.

Border cells migrate as a morphologically distinct and interconnected group. Knockdown of *CG6509* in border cells disrupted this cluster organization in addition to delaying their migration. This suggests that CG6509 helps keep border cells together in a collective cluster. The predicted mammalian homolog of CG6509, Dlg5, has been implicated in regulating cell migration (Smolen *et al.* 2010) and epithelial polarity (Nechiporuk *et al.* 2007). From mouse knockout studies, Dlg5 was proposed to maintain cell polarity through trafficking of cadherin-catenin complexes and stabilization of adherens junctions (Nechiporuk *et al.* 2007). The cluster morphology defects we observed with *CG6509* knockdown are consistent with defects in cell polarity and/or cell-cell adhesion. Nonetheless, we did not observe gross alterations in the levels or localization of E-cadherin and polarity proteins in *CG6509* RNAi border cells; however, we cannot rule out the possibility of subtle changes in these proteins and/or residual *CG6509* gene function. The disorganized cluster phenotypes produced by knockdown of *CG6509* resemble those caused by loss of JNK activity (Llense and Martín-Blanco 2008; Melani *et al.* 2008). JNK signaling promotes border cell cluster cohesion through regulation of cell polarity proteins such as Baz and cell-cell adhesion via Integrins and E-cadherin (Llense and Martín-Blanco 2008). Further investigation will be needed to determine whether CG6509 regulates cell-cell contacts within the border cell cluster and whether it functions downstream of or in parallel to JNK signaling.

## Supplementary Material

Supporting Information

## References

[bib1] Abdelilah-Seyfried S. Cox D. N. Jan Y. N., 2003 Bazooka is a permissive factor for the invasive behavior of discs large tumor cells in Drosophila ovarian follicular epithelia. Development 130: 1927–1935 12642496 10.1242/dev.00420

[bib2] Ashburner M. Ball C. A. Blake J. A. Botstein D. Butler H., 2000 Gene ontology: tool for the unification of biology. The Gene Ontology Consortium. Nat. Genet. 25: 25–29 10802651 10.1038/75556 PMC3037419

[bib3] Bai J. Uehara Y. Montell D. J., 2000 Regulation of invasive cell behavior by taiman, a Drosophila protein related to AIB1, a steroid receptor coactivator amplified in breast cancer. Cell 103: 1047–1058 11163181 10.1016/s0092-8674(00)00208-7

[bib4] Bastock R. Strutt D., 2007 The planar polarity pathway promotes coordinated cell migration during Drosophila oogenesis. Development 134: 3055–3064 17652348 10.1242/dev.010447 PMC1991286

[bib5] Beccari S. Teixeira L. Rørth P., 2002 The JAK/STAT pathway is required for border cell migration during Drosophila oogenesis. Mech. Dev. 111: 115–123 11804783 10.1016/s0925-4773(01)00615-3

[bib6] Bernard O., 2007 Lim kinases, regulators of actin dynamics. Int. J. Biochem. Cell Biol. 39: 1071–1076 17188549 10.1016/j.biocel.2006.11.011

[bib7] Bianco A. Poukkula M. Cliffe A. Mathieu J. Luque C. M., 2007 Two distinct modes of guidance signalling during collective migration of border cells. Nature 448: 362–365 17637670 10.1038/nature05965

[bib8] Bilder D., 2001 PDZ proteins and polarity: functions from the fly. Trends Genet. 17: 511–519 11525834 10.1016/s0168-9525(01)02407-6

[bib9] Booker M. Samsonova A. A. Kwon Y. Flockhart I. Mohr S. E., 2011 False negative rates in Drosophila cell-based RNAi screens: a case study. BMC Genomics 12: 50 21251254 10.1186/1471-2164-12-50 PMC3036618

[bib10] Borghese L. Fletcher G. Mathieu J. Atzberger A. Eades W. C., 2006 Systematic analysis of the transcriptional switch inducing migration of border cells. Dev. Cell 10: 497–508 16580994 10.1016/j.devcel.2006.02.004 PMC2955450

[bib11] Brand A. H. Perrimon N., 1993 Targeted gene expression as a means of altering cell fates and generating dominant phenotypes. Development 118: 401–415 8223268 10.1242/dev.118.2.401

[bib13] Cox D. N. Seyfried S. A. Jan L. Y. Jan Y. N., 2001 Bazooka and atypical protein kinase C are required to regulate oocyte differentiation in the Drosophila ovary. Proc. Natl. Acad. Sci. USA 98: 14475–14480 11734648 10.1073/pnas.261565198 PMC64706

[bib14] Cronin S. J. F. Nehme N. T. Limmer S. Liegeois S. Pospisilik J. A., 2009 Genome-wide RNAi screen identifies genes involved in intestinal pathogenic bacterial infection. Science 325: 340–343 19520911 10.1126/science.1173164 PMC2975362

[bib15] Dietzl G. Chen D. Schnorrer F. Su K.-C. Barinova Y., 2007 A genome-wide transgenic RNAi library for conditional gene inactivation in Drosophila. Nature 448: 151–156 17625558 10.1038/nature05954

[bib16] Dow L. E. Humbert P. O., 2007 Polarity regulators and the control of epithelial architecture, cell migration, and tumorigenesis. Int. Rev. Cytol. 262: 253–302 17631191 10.1016/S0074-7696(07)62006-3

[bib17] Duchek P. Rørth P., 2001 Guidance of cell migration by EGF receptor signaling during Drosophila oogenesis. Science 291: 131–133 11141565 10.1126/science.291.5501.131

[bib18] Duchek P. Somogyi K. Jékely G. Beccari S. Rørth P., 2001 Guidance of cell migration by the Drosophila PDGF/VEGF receptor. Cell 107: 17–26 11595182 10.1016/s0092-8674(01)00502-5

[bib19] Etienne-Manneville S., 2008 Polarity proteins in migration and invasion. Oncogene 27: 6970–6980 19029938 10.1038/onc.2008.347

[bib20] Friedl P. Gilmour D., 2009 Collective cell migration in morphogenesis, regeneration and cancer. Nat. Rev. Mol. Cell Biol. 10: 445–457 19546857 10.1038/nrm2720

[bib21] Friedl P. Locker J. Sahai E. Segall J. E., 2012 Classifying collective cancer cell invasion. Nat. Cell Biol. 14: 777–783 22854810 10.1038/ncb2548

[bib22] Fulga T. A. Rørth P., 2002 Invasive cell migration is initiated by guided growth of long cellular extensions. Nat. Cell Biol. 4: 715–719 12198500 10.1038/ncb848

[bib23] Geisbrecht E. R. Haralalka S. Swanson S. K. Florens L. Washburn M. P., 2008 Drosophila ELMO/CED-12 interacts with Myoblast city to direct myoblast fusion and ommatidial organization. Dev. Biol. 314: 137–149 18163987 10.1016/j.ydbio.2007.11.022 PMC2697615

[bib24] Ghiglione C. Devergne O. Georgenthum E. Carballès F. Médioni C., 2002 The Drosophila cytokine receptor Domeless controls border cell migration and epithelial polarization during oogenesis. Development 129: 5437–5447 12403714 10.1242/dev.00116

[bib25] Goode S. Perrimon N., 1997 Inhibition of patterned cell shape change and cell invasion by Discs large during Drosophila oogenesis. Genes Dev. 11: 2532–2544 9334318 10.1101/gad.11.19.2532 PMC316565

[bib26] Guillemot L. Paschoud S. Pulimeno P. Foglia A. Citi S., 2008 The cytoplasmic plaque of tight junctions: a scaffolding and signalling center. Biochim. Biophys. Acta 1778: 601–613 18339298 10.1016/j.bbamem.2007.09.032

[bib27] Gustafson K. Boulianne G. L., 1996 Distinct expression patterns detected within individual tissues by the GAL4 enhancer trap technique. Genome 39: 174–182 8851804 10.1139/g96-023

[bib28] Harris B. Z. Lim W. A., 2001 Mechanism and role of PDZ domains in signaling complex assembly. J. Cell Sci. 114: 3219–3231 11591811 10.1242/jcs.114.18.3219

[bib29] Hidalgo-Carcedo C. Hooper S. Chaudhry S. I. Williamson P. Harrington K., 2011 Collective cell migration requires suppression of actomyosin at cell-cell contacts mediated by DDR1 and the cell polarity regulators Par3 and Par6. Nat. Cell Biol. 13: 49–58 21170030 10.1038/ncb2133 PMC3018349

[bib30] Higuchi N. Kohno K. Kadowaki T., 2009 Specific retention of the protostome-specific PsGEF may parallel with the evolution of mushroom bodies in insect and lophotrochozoan brains. BMC Biol. 7: 21 19422675 10.1186/1741-7007-7-21 PMC2684095

[bib31] Huelsmann S. S. Hepper C. C. Marchese D. D. Knöll C. C. Reuter R. R., 2006 The PDZ-GEF dizzy regulates cell shape of migrating macrophages via Rap1 and integrins in the Drosophila embryo. Development 133: 2915–2924 16818452 10.1242/dev.02449

[bib32] Humbert P. O. Dow L. E. Russell S. M., 2006 The Scribble and Par complexes in polarity and migration: friends or foes? Trends Cell Biol. 16: 622–630 17067797 10.1016/j.tcb.2006.10.005

[bib33] Humbert P. O. Grzeschik N. A. Brumby A. M. Galea R. Elsum I., 2008 Control of tumourigenesis by the Scribble/Dlg/Lgl polarity module. Oncogene 27: 6888–6907 19029932 10.1038/onc.2008.341

[bib34] Huynh J. R. Petronczki M. Knoblich J. A. St Johnston D., 2001 Bazooka and PAR-6 are required with PAR-1 for the maintenance of oocyte fate in Drosophila. Curr. Biol. 11: 901–906 11516655 10.1016/s0960-9822(01)00244-5

[bib35] Kaverina I. Straube A., 2011 Regulation of cell migration by dynamic microtubules. Semin. Cell Dev. Biol. 22: 968–974 22001384 10.1016/j.semcdb.2011.09.017 PMC3256984

[bib36] Kim J. H. Cho A. Yin H. Schafer D. A. Mouneimne G., 2011 Psidin, a conserved protein that regulates protrusion dynamics and cell migration. Genes Dev. 25: 730–741 21406550 10.1101/gad.2028611 PMC3070935

[bib37] Kim S. Y. Renihan M. K. Boulianne G. L., 2006 Characterization of big bang, a novel gene encoding for PDZ domain-containing proteins that are dynamically expressed throughout Drosophila development. Gene Expr. Patterns 6: 504–518 16423565 10.1016/j.modgep.2005.10.009

[bib38] Laflamme C. Assaker G. Ramel D. Dorn J. F. She D., 2012 Evi5 promotes collective cell migration through its Rab-GAP activity. J. Cell Biol. 198: 57–67 22778279 10.1083/jcb.201112114 PMC3392932

[bib39] Laprise P. Tepass U., 2011 Novel insights into epithelial polarity proteins in Drosophila. Trends Cell Biol. 21: 401–408 21530265 10.1016/j.tcb.2011.03.005

[bib40] Liu Y. Montell D. J., 1999 Identification of mutations that cause cell migration defects in mosaic clones. Development 126: 1869–1878 10101121 10.1242/dev.126.9.1869

[bib41] Llense F. Martín-Blanco E., 2008 JNK signaling controls border cell cluster integrity and collective cell migration. Curr. Biol. 18: 538–544 18394890 10.1016/j.cub.2008.03.029

[bib42] Martin-Belmonte F. Perez-Moreno M., 2012 Epithelial cell polarity, stem cells and cancer. Nat. Rev. Cancer 12: 23–38 22169974 10.1038/nrc3169

[bib43] Mathieu J. Sung H.-H. Pugieux C. Soetaert J. Rørth P., 2007 A sensitized PiggyBac-based screen for regulators of border cell migration in Drosophila. Genetics 176: 1579–1590 17483425 10.1534/genetics.107.071282 PMC1931525

[bib44] McDonald J. A. Montell D. J., 2005 Analysis of cell migration using Drosophila as a model system. Methods Mol. Biol. 294: 175–202 15576913 10.1385/1-59259-860-9:175

[bib45] McDonald J. A. Pinheiro E. M. Montell D. J., 2003 PVF1, a PDGF/VEGF homolog, is sufficient to guide border cells and interacts genetically with Taiman. Development 130: 3469–3478 12810594 10.1242/dev.00574

[bib46] McDonald J. A. Pinheiro E. M. Kadlec L. Schupbach T. Montell D. J., 2006 Multiple EGFR ligands participate in guiding migrating border cells. Dev. Biol. 296: 94–103 16712835 10.1016/j.ydbio.2006.04.438

[bib47] McDonald J. A. Khodyakova A. Aranjuez G. Dudley C. Montell D. J., 2008 PAR-1 kinase regulates epithelial detachment and directional protrusion of migrating border cells. Curr. Biol. 18: 1659–1667 18976916 10.1016/j.cub.2008.09.041 PMC2593744

[bib48] Melani M. Simpson K. J. Brugge J. S. Montell D., 2008 Regulation of cell adhesion and collective cell migration by hindsight and its human homolog RREB1. Curr. Biol. 18: 532–537 18394891 10.1016/j.cub.2008.03.024

[bib49] Montell D. J., 2003 Border-cell migration: the race is on. Nat. Rev. Mol. Cell Biol. 4: 13–24 12511865 10.1038/nrm1006

[bib50] Montell D. J. Rørth P. Spradling A. C., 1992 slow border cells, a locus required for a developmentally regulated cell migration during oogenesis, encodes Drosophila C/EBP. Cell 71: 51–62 1394432 10.1016/0092-8674(92)90265-e

[bib51] Mummery-Widmer J. L. Yamazaki M. Stoeger T. Novatchkova M. Bhalerao S., 2009 Genome-wide analysis of Notch signalling in Drosophila by transgenic RNAi. Nature 458: 987–992 19363474 10.1038/nature07936 PMC2988197

[bib52] Murphy A. M. Montell D. J., 1996 Cell type-specific roles for Cdc42, Rac, and RhoL in Drosophila oogenesis. J. Cell Biol. 133: 617–630 8636236 10.1083/jcb.133.3.617 PMC2120826

[bib53] Nakayama M. Goto T. M. Sugimoto M. Nishimura T. Shinagawa T., 2008 Rho-kinase phosphorylates PAR-3 and disrupts PAR complex formation. Dev. Cell 14: 205–215 18267089 10.1016/j.devcel.2007.11.021

[bib54] Nechiporuk T. Fernandez T. E. Vasioukhin V., 2007 Failure of epithelial tube maintenance causes hydrocephalus and renal cysts in Dlg5−/− mice. Dev. Cell 13: 338–350 17765678 10.1016/j.devcel.2007.07.017 PMC2023971

[bib55] Ni J.-Q. Liu L.-P. Binari R. Hardy R. Shim H.-S., 2009 A Drosophila resource of transgenic RNAi lines for neurogenetics. Genetics 182: 1089–1100 19487563 10.1534/genetics.109.103630 PMC2728850

[bib56] Ni J.-Q. Zhou R. Czech B. Liu L.-P. Holderbaum L., 2011 A genome-scale shRNA resource for transgenic RNAi in Drosophila. Nat. Methods 8: 405–407 21460824 10.1038/nmeth.1592 PMC3489273

[bib57] Niewiadomska P. Godt D. Tepass U., 1999 DE-Cadherin is required for intercellular motility during Drosophila oogenesis. J. Cell Biol. 144: 533–547 9971747 10.1083/jcb.144.3.533 PMC2132905

[bib58] Nishita M. Tomizawa C. Yamamoto M. Horita Y. Ohashi K., 2005 Spatial and temporal regulation of cofilin activity by LIM kinase and Slingshot is critical for directional cell migration. J. Cell Biol. 171: 349–359 16230460 10.1083/jcb.200504029 PMC2171197

[bib59] Oliva C. Escobedo P. Astorga C. Molina C. Sierralta J., 2012 Role of the MAGUK protein family in synapse formation and function. Dev. Neurobiol. 72: 57–72 21739617 10.1002/dneu.20949

[bib60] Pagliarini R. A. Xu T., 2003 A genetic screen in Drosophila for metastatic behavior. Science 302: 1227–1231 14551319 10.1126/science.1088474

[bib61] Perrimon N. Ni J.-Q. Perkins L., 2010 In vivo RNAi: today and tomorrow. Cold Spring Harb. Perspect. Biol. 2: a003640 20534712 10.1101/cshperspect.a003640 PMC2908776

[bib62] Pinheiro E. M. Montell D. J., 2004 Requirement for Par-6 and Bazooka in Drosophila border cell migration. Development 131: 5243–5251 15456726 10.1242/dev.01412

[bib63] Prasad M. Montell D. J., 2007 Cellular and molecular mechanisms of border cell migration analyzed using time-lapse live-cell imaging. Dev. Cell 12: 997–1005 17543870 10.1016/j.devcel.2007.03.021

[bib64] Prasad M. Jang A. C.-C. Starz-Gaiano M. Melani M. Montell D. J., 2007 A protocol for culturing Drosophila melanogaster stage 9 egg chambers for live imaging. Nat. Protoc. 2: 2467–2473 17947988 10.1038/nprot.2007.363

[bib65] Queenan A. M. Ghabrial A. Schüpbach T., 1997 Ectopic activation of torpedo/Egfr, a Drosophila receptor tyrosine kinase, dorsalizes both the eggshell and the embryo. Development 124: 3871–3880 9367443 10.1242/dev.124.19.3871

[bib66] Ranganathan R. Ross E. M., 1997 PDZ domain proteins: scaffolds for signaling complexes. Curr. Biol. 7: R770–R773 9382826 10.1016/s0960-9822(06)00401-5

[bib67] Ridley A. J., 2011 Life at the leading edge. Cell 145: 1012–1022 21703446 10.1016/j.cell.2011.06.010

[bib68] Rørth P. Szabo K. Bailey A. Laverty T. Rehm J., 1998 Systematic gain-of-function genetics in Drosophila. Development 125: 1049–1057 9463351 10.1242/dev.125.6.1049

[bib69] Royer C. Lu X., 2011 Epithelial cell polarity: a major gatekeeper against cancer? Cell Death Differ. 18: 1470–1477 21617693 10.1038/cdd.2011.60 PMC3178423

[bib70] Schnorrer F. Schönbauer C. Langer C. C. H. Dietzl G. Novatchkova M., 2010 Systematic genetic analysis of muscle morphogenesis and function in Drosophila. Nature 464: 287–291 20220848 10.1038/nature08799

[bib71] Shin K. Wang Q. Margolis B., 2007 PATJ regulates directional migration of mammalian epithelial cells. EMBO Rep. 8: 158–164 17235357 10.1038/sj.embor.7400890 PMC1796763

[bib72] Sierralta J. Mendoza C., 2004 PDZ-containing proteins: alternative splicing as a source of functional diversity. Brain Res. Brain Res. Rev. 47: 105–115 15572166 10.1016/j.brainresrev.2004.06.002

[bib73] Silver D. L. Montell D. J., 2001 Paracrine signaling through the JAK/STAT pathway activates invasive behavior of ovarian epithelial cells in Drosophila. Cell 107: 831–841 11779460 10.1016/s0092-8674(01)00607-9

[bib74] Silver D. L. Geisbrecht E. R. Montell D. J., 2005 Requirement for JAK/STAT signaling throughout border cell migration in Drosophila. Development 132: 3483–3492 16000386 10.1242/dev.01910

[bib75] Simpson K. J. Selfors L. M. Bui J. Reynolds A. Leake D., 2008 Identification of genes that regulate epithelial cell migration using an siRNA screening approach. Nat. Cell Biol. 10: 1027–1038 19160483 10.1038/ncb1762

[bib76] Smolen G. A. Zhang J. Zubrowski M. J. Edelman E. J. Luo B., 2010 A genome-wide RNAi screen identifies multiple RSK-dependent regulators of cell migration. Genes Dev. 24: 2654–2665 21062900 10.1101/gad.1989110 PMC2994039

[bib77] Subbaiah V. K. Kranjec C. Thomas M. Banks L., 2011 PDZ domains: the building blocks regulating tumorigenesis. Biochem. J. 439: 195–205 21954943 10.1042/BJ20110903

[bib78] Szafranski P. Goode S., 2004 A Fasciclin 2 morphogenetic switch organizes epithelial cell cluster polarity and motility. Development 131: 2023–2036 15056617 10.1242/dev.01097

[bib79] Szafranski P. Goode S., 2007 Basolateral junctions are sufficient to suppress epithelial invasion during Drosophila oogenesis. Dev. Dyn. 236: 364–373 17103414 10.1002/dvdy.21020

[bib80] Thomas P. D. Kejariwal A. Campbell M. J. Mi H. Diemer K., 2003 PANTHER: a browsable database of gene products organized by biological function, using curated protein family and subfamily classification. Nucleic Acids Res. 31: 334–341 12520017 10.1093/nar/gkg115 PMC165562

[bib81] Tonikian R. Zhang Y. Sazinsky S. L. Currell B. Yeh J.-H., 2008 A specificity map for the PDZ domain family. PLoS Biol. 6: e239 18828675 10.1371/journal.pbio.0060239 PMC2553845

[bib82] Van de Bor V. Zimniak G. Cerezo D. Schaub S. Noselli S., 2011 Asymmetric localisation of cytokine mRNA is essential for JAK/STAT activation during cell invasiveness. Development 138: 1383–1393 21350010 10.1242/dev.056184

[bib83] Vinkemeier U., 2004 Getting the message across, STAT! Design principles of a molecular signaling circuit. J. Cell Biol. 167: 197–201 15504906 10.1083/jcb.200407163 PMC2172545

[bib85] Wang X. Bo J. Bridges T. Dugan K. D. Pan T.-C., 2006 Analysis of cell migration using whole-genome expression profiling of migratory cells in the Drosophila ovary. Dev. Cell 10: 483–495 16580993 10.1016/j.devcel.2006.02.003

[bib86] Xi R. McGregor J. R. Harrison D. A., 2003 A gradient of JAK pathway activity patterns the anterior-posterior axis of the follicular epithelium. Dev. Cell 4: 167–177 12586061 10.1016/s1534-5807(02)00412-4

[bib87] Yang N. Inaki M. Cliffe A. Rørth P., 2012 Microtubules and Lis-1/NudE/Dynein regulate invasive cell-on-cell migration in Drosophila. PLoS ONE 7: e40632 22808215 10.1371/journal.pone.0040632 PMC3396602

[bib88] Yilmaz M. Christofori G., 2010 Mechanisms of motility in metastasizing cells. Mol. Cancer Res. 8: 629–642 20460404 10.1158/1541-7786.MCR-10-0139

[bib89] Zhang L. Luo J. Wan P. Wu J. Laski F., 2011 Regulation of cofilin phosphorylation and asymmetry in collective cell migration during morphogenesis. Development 138: 455–464 21205790 10.1242/dev.046870

