# Supplementary material for: On the Role of PDZ Domain-Encoding Genes in Drosophila Border Cell Migration
Source: G3 (Bethesda). 2012 Nov 1;2(11):1379–91. doi: 10.1534/g3.112.004093 (PMC3484668; doi:10.1534/g3.112.004093)
Supplement: Supporting Information [file supp_2_11_1379__index.html]

Supporting Information 

# On the Role of PDZ Domain-Encoding Genes in Drosophila Border Cell Migration

## Supporting Information for Aranjuez *et al.*, 2012

**Files in this Data Supplement:**

- Supporting Information - Figures S1-S4 and Table S1 (PDF, 2.2 MB)
- Figure S1 - Drosophila PDZ-domain-containing proteins are functionally diverse and involved in numerous processes (PDF, 494 KB)
- Figure S2 - *slbo*-GAL4-driven RNAi expression targeting three positive candidates (PDF, 277 KB)
- Figure S3 - Gene expression levels in control and RNAi knockdown measured by quantitative RT-PCR (PDF, 330 KB)
- Figure S4 - Bbg function in early development of border cells (PDF, 1.2 MB)
- Table S1 - Complete results of the PDZ RNAi survey of border cell migration (PDF, 116 KB)
