# Supplementary material for: On the Role of PDZ Domain-Encoding Genes in Drosophila Border Cell Migration
Source: G3 (Bethesda). 2012 Nov 1;2(11):1379–91. doi: 10.1534/g3.112.004093 (PMC3484668; doi:10.1534/g3.112.004093)
Supplement: Supporting Information [file supp_2.11.1379_004093SI.pdf]

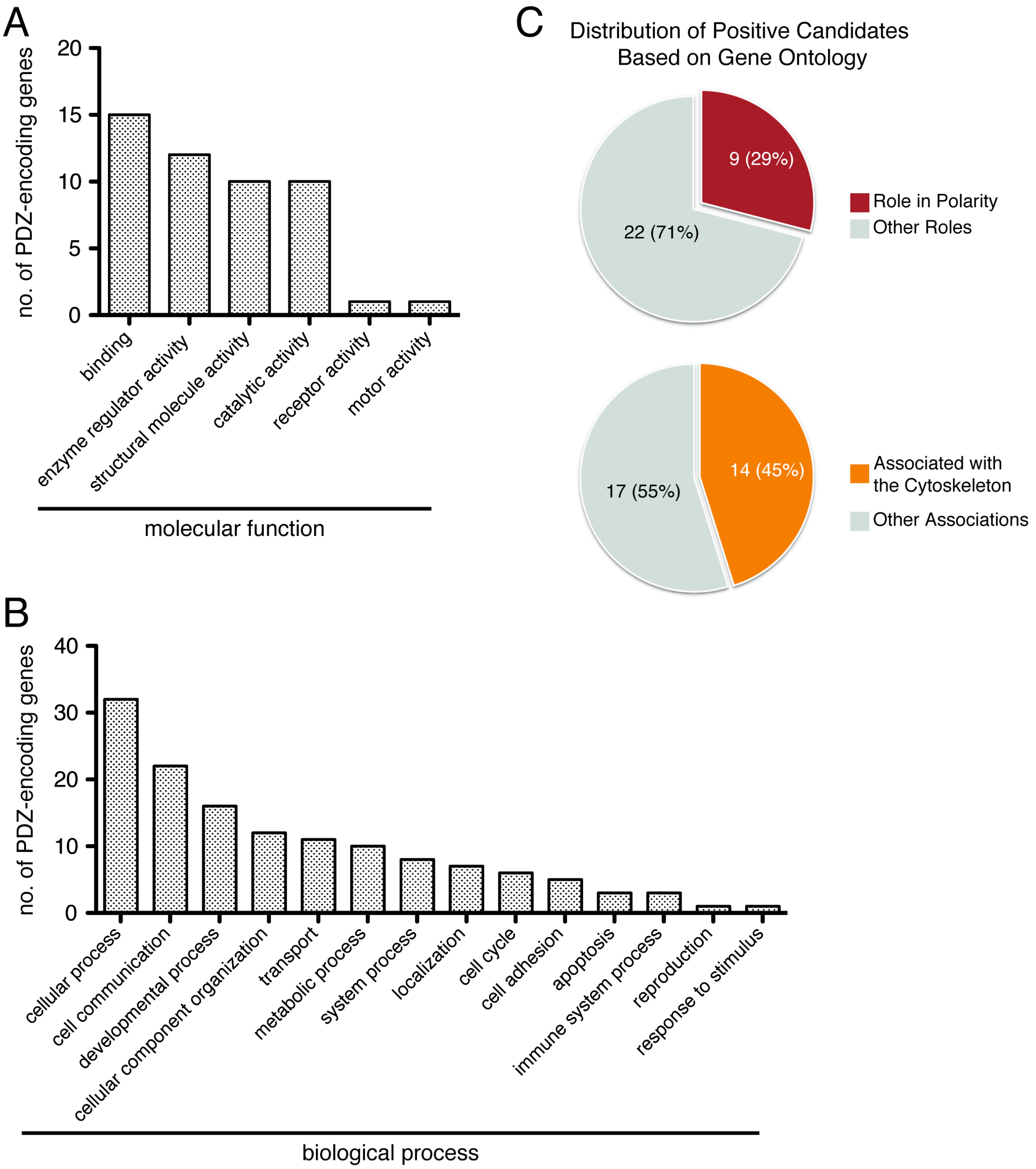

Figure S1 Drosophila PDZ-domain-containing proteins are functionally diverse and involved in numerous processes. (A-B) Of the 66 genes annotated to contain a PDZ domain, 51 were analyzed by PANTHER (http://www.pantherdb.org). (A) PDZ proteins are found in almost half (6/14) of the classifications for molecular function and predominantly annotated as having binding, enzyme regulator, structural, or catalytic activity. Molecular function is defined as the action that a protein performs on its direct target. A protein is assigned only one molecular function. (B) PDZ proteins contribute to 14 out of 19 annotated biological processes. A protein can contribute to multiple processes. (C) Positive hit genes were surveyed for roles in polarity or association with the cytoskeleton using AmiGO (http://amigo.geneontology.org). Genes can fall into both categories. The polarity genes include: *baz, CASK, dsh, Lap1, par-6, Patj, pyd, sdt* and *veli*. The following genes are associated with the cytoskeleton: *CG14168, CG42319, CG42788, CG5921, CG6498, dsh, Lap1, LIMK1, Patj, Ptpmeg, RhoGAP19D, Syn2, veli,* and *Zasp52*.

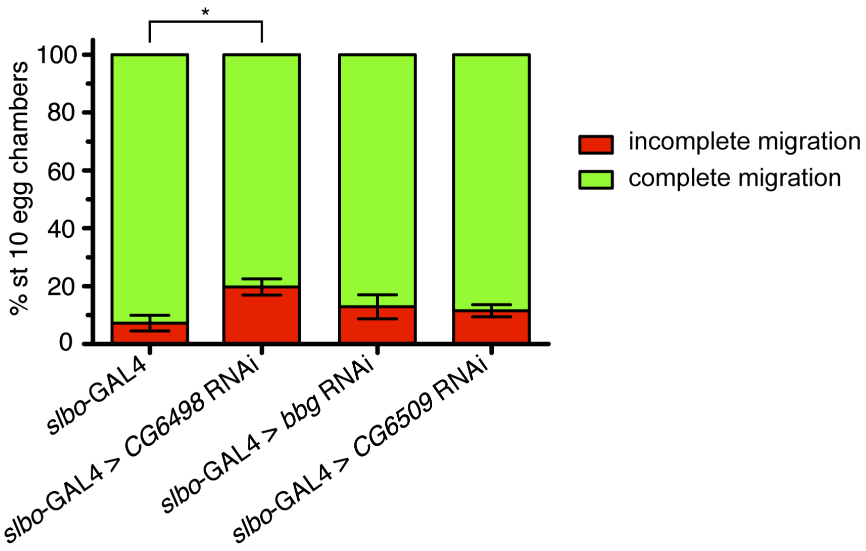

Figure S2 *slbo*-GAL4-driven RNAi expression targeting three positive candidates. Quantification of border cell migration at stage 10, shown as the percentage of border cells with complete (green) or incomplete (red) migration in egg chambers expressing *CG6498* RNAi v35100, *bbg* RNAi v15975, and *CG6509* RNAi v22496 in border cells using *slbo*-GAL4. Two-tailed unpaired t-test was used to determine statistical significance (*, $p < 0.05$). At least 50 egg chambers were scored in at least three trials.

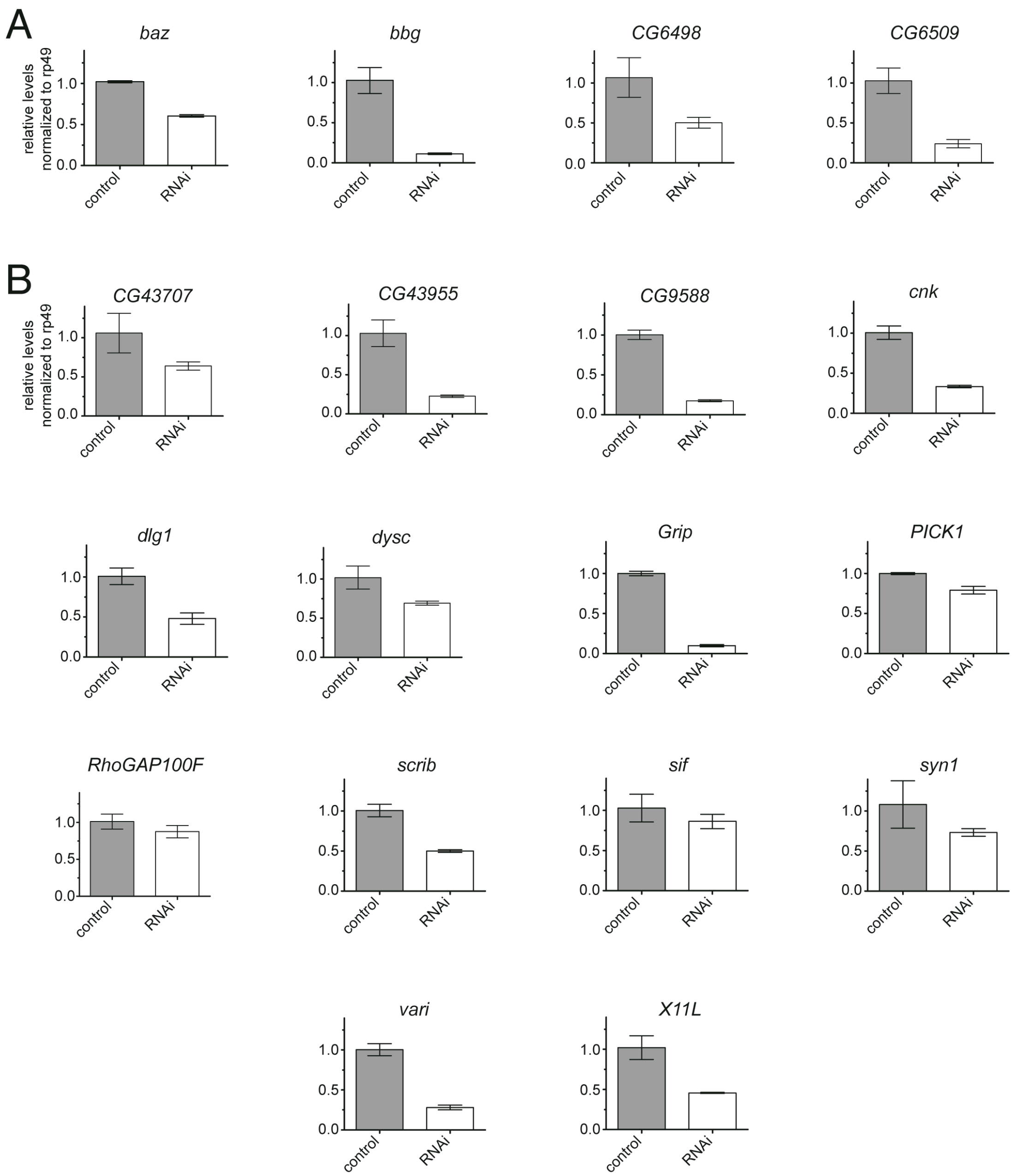

Figure S3 Gene expression levels in control and RNAi knockdown measured by quantitative RT-PCR. Ubiquitous expression of RNAi against a subset of (A) positive candidates and (B) negative candidates result in varying degrees of knockdown. The mean transcript levels were calculated from three biological replicates using $\Delta\Delta C_T$ method and normalized to *rp49* mRNA levels. Controls are RNAi to GFP. Error bars represent standard deviation.

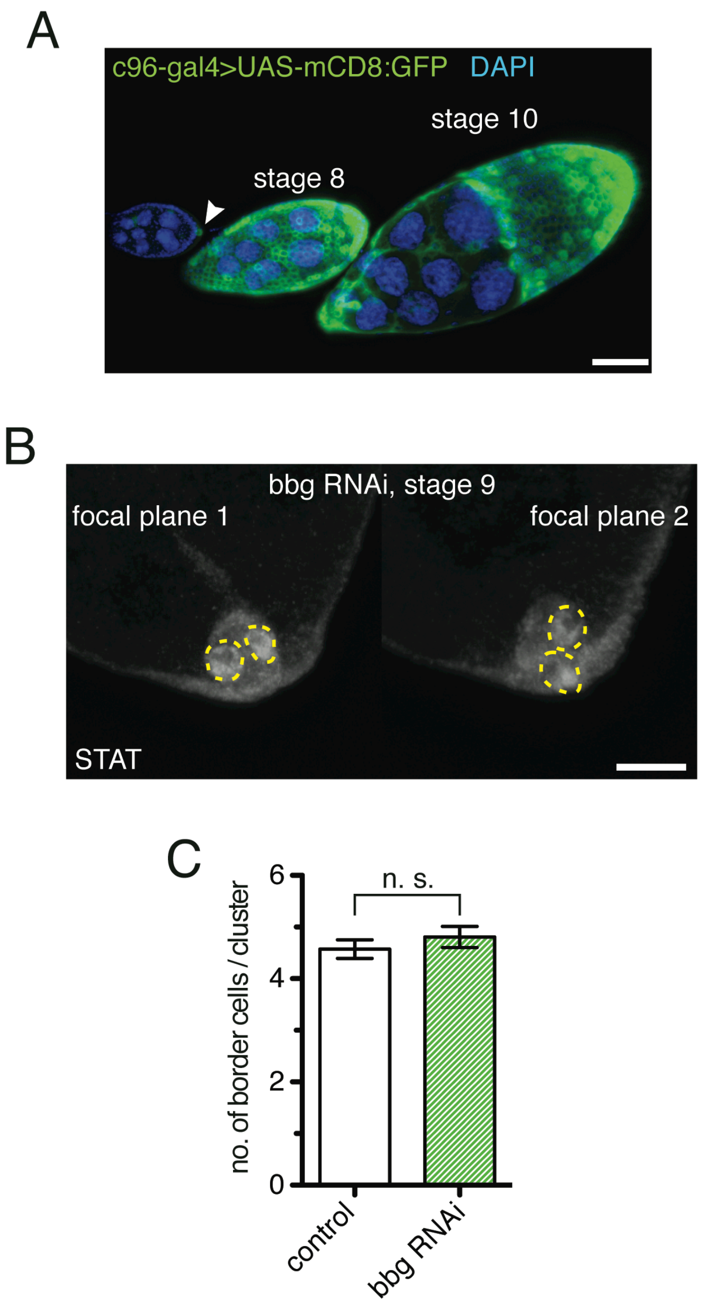

Figure S4 Bbg function in early development of border cells. (A) *c96*-GAL4 expression (green) in egg chambers is limited to the polar cells (arrowhead) in the early stages but expands to most of the follicle cell epithelium starting at stage 8 of Drosophila oogenesis. The image was acquired to show the follicle cell epithelium. The nuclei are visualized with DAPI (blue). Scale bar is 50 μm. (B) STAT nuclear enrichment (yellow dashed circles) is observed in border cells expressing *bbg* RNAi line v15975 driven by *c306*-GAL4 before detachment (stage 9). Upon *bbg* knockdown, 72% (N=21) of detaching clusters still retain nuclear STAT enrichment in all or some border cells in the cluster. The images are of the same border cell cluster taken at different focal planes. Scale bar is 10 μm. (C) Border cell recruitment is unaffected by *bbg* RNAi. On average, both control (N=35) and bbg RNAi (N=31) clusters have ~5 border cells. Error bars represent SEM. N.S., not statistically significant (two-tailed unpaired t-test).

Table S1 Complete results of the PDZ RNAi survey of border cell migration

Group[a]	Gene Name	Gene Symbol	Annotation Symbol	RNAi line	trial 1		trial 2		trial 3	
					% defect[b]	n[c]	% defect	n	% defect	n
Pos. Mult.	bazooka	baz	CG5055	2914 (M)[d]	43%[e]	116				
				2915 (M)	23%	137				
Pos. Mult.	big bang	bbg	CG42230	15975	19%	58	15%	112		
				101691	10%	132				
				36111	6%	78				
Pos. Mult.	CASK ortholog	CASK	CG6703	34185 (S†,M)[f]	23%	79	15%	80		
				34184	22%	122	10%	251		
				104793	2%	139				
Pos. Mult.	CG5921	CG5921	CG5921	37875	33%	94	23%	106		
				37874	18%	91				
Pos. Mult.	CG6498	CG6498	CG6498	35101	20%	97	11%	125		
				35100	17%	116	12%	164		
				109282	13%	176				
Pos. Mult.	CG6509	CG6509	CG6509	46234 (S†,M)	11%	54	12%	139		
				101596	11%	74				
				22496 (S†,M)	9%	87				
Pos. Mult.	Gef26	Gef26	CG9491	27017 (M)	19%	86	28%	197		
				27015 (M)	10%	98	20%	98		
				105159	15%	59				
Pos. Mult.	Lap1	Lap1	CG10255	18600 (M)	15%	112	13%	119		
				JF02362[g]	10%	134				
Pos. Mult.	LIM-kinase1	LIMK1	CG1848	25344	10%	125	8%	194	12%	220
				25343	6%	128				
				2x RNAi[h]	18%	50				
Pos. Mult.	par-6	par-6	CG5884	19732 (M†)	38%	58	26%	114		
				108560	44%	59				
				2x RNAi[i]	14%	132				
Pos. Mult.	PatJ	Patj	CG12021	31620	13%	168	20%	128		
				12021R-3[j]	26%	184				
				101877	3%	63				
				12021R-4[j]	lethal		lethal			
Pos. Mult.	Rim	Rim	CG33547	39384	26%	92	12%	50		
				39385	24%	94	1%	78	12%	113
				JF02610[g]	7%	254				
Pos. Mult.	stardust	sdt	CG32717	100685	39%	62	11%	57		
				29844	13%	182				
				29843	6%	197				
Pos. Mult.	veli	veli	CG7662	43094 (M†)	50%	88	27%	101		

				46963 (M)	31%	61				
Pos. Single	CG14168	CG14168	CG14168	17414	13%	55	21%	226		
				103225	2%	56				
Pos. Single	CG34375	CG34375	CG34375	23904	29%	63	5%	166	13%	143
				102586	4%	56				
Pos. Single	CG42319	CG42319	CG42319	23928	41%	152				
				32835 (M)	8%	197				
Pos. Single	CG42788	CG42788	CG42788	22311	40%	72	5%	134	11%	196
				108180	1%	123				
				45034	4%	72				
Pos. Single	CG6688	CG6688	CG6688	48260	15%	86	19%	214		
				106732	1%	139				
Pos. Single	Dishevelled	dsh	CG18361	101525	12%	93				
				JF01254[g]	6%	188				
				18361R-2[j]	lethal					
				18361R-3[j]	lethal					
				JF01253[g]	8%	89				
Pos. Single	Efa6 pleckstrin and Sec7 domain containing	Efa6	CG31158	42321	14%	128	12%	109		
Pos. Single	HtrA2	HtrA2	CG8464	24104	16%	75	9%	153	19%	260
				24106	9%	132				
	Magi	Magi	CG30388	41735	13%	128	4%	167		
				41736	10%	62				
Pos. Single	menage a trois	metro	CG30021	29967	10%	73				
				29965	1%	128				
				110814	1%	129				
Pos. Single	polychaetoid	pyd	CG43140	38863	15%	111	23%	188		
				104159	6%	142				
Pos. Single	Ptpmeg	Ptpmeg	CG1228	38651	12%	165	22%	78		
				103740	3%	130				
Pos. Single	RhoGAP19D	RhoGAP19D	CG1412	43955 (M†)	42%	57	59%	165		
				HMS00352[g]	9%	161				
				dsRNA 2.1[k]	8%	217				
				dsRNA 3.1[k]	3%	62				
Pos. Single	Rhophilin	Rhp	CG8497	24111	13%	102	11%	204		
				110377	3%	152				
Pos. Single	Slip1	Slip1	CG1783	33006 (S)	22%	124	10%	168		
				101106	6%	71				
Pos. Single	Syntrophin-like 2	Syn2	CG4905	JF02999[g]	11%	157	6%	84		
				110602	10%	67				
Pos. Single	Z band	Zasp52	CG30084	36563 (S†,M†)	37%	97	19%	124		

	alternatively spliced PDZ-motif protein 52							
				106177	3%	65		
				JF01133[g]	4%	227		
Negative	canoe	cno	CG42312	102686	3%	92		
Negative	CG10362	CG10362	CG10362	8317	4%	52		
Negative	CG15617	CG15617	CG15617	19148	37%	65	7%	162
				19149	7%	94	6%	167
				103267	2%	50		
Negative	CG15803	CG15803	CG15803	43635	3%	59		
				43636	1%	135		
Negative	CG32758	CG32758	CG32758	28457	4%	99		
				108542	4%	130		
Negative	CG3402	CG3402	CG3402	21485 (M†)	7%	58		
				110191	6%	125		
Negative	CG43707	CG43707	CG43707	25846S	8%	130		
				25847 (S†)	6%	89		
Negative	CG43955	CG43955	CG43955	103267	2%	50		
Negative	CG6619	CG6619	CG6619	15622	4%	67		
Negative	CG9588	CG9588	CG9588	47763	2%	95		
				100126	4%	167		
				HM05013[g]	0%	93		
Negative	connector enhancer of kar	cnk	CG6556	107746	3%	135		
				HMS00238[g]	3%	152		
Negative	discs large 1	dlg1	CG1725	41134 (S†,M†)	4%	85		
				109274	2%	132		
				41136	lethal			
				HMS01521[g]	lethal			
Negative	dyschronic	dysc	CG43749	23278	9%	92		
				14082	5%	59		
				110019	3%	135		
Negative	Glutamate receptor binding protein	Grip	CG14447	21003 (S†)	5%	57		
				103551	6%	71		
Negative	Grasp65	Grasp65	CG7809	22564	1%	125		
Negative	inactivation no afterpotential D	inaD	CG3504	26211	6%	64		
Negative	kermit	kermit	CG11546	109297	2%	131		
Negative	locomotion defects	loco	CG5248	9248 (M)	10%	67	6%	237

				110275	7%	149		
				HMS00455[g]	3%	189		
Negative	PICK1	PICK1	CG6167	22268	4%	74		
				104486	1%	163		
				JF01199[g]	3%	222		
Negative	Prosap	Prosap	CG30483	21216 (M†)	5%	59		
				103592	4%	98		
Negative	Protostome-specific GEF	PsGEF	CG43947	32088	8%	122		
				109769	1%	154		
Negative	RhoGAP100F	RhoGAP100F	CG1976	106241	0%	62		
				HMS00740[g]	1%	161		
Negative	RhoGEF2	RhoGEF2	CG9635	JF01747[g]	13%	145	6%	135
				HMS01118[g]	7%	180		
				110577	3%	90		
Negative	scribbled	scrib	CG43398	105412	5%	172		
				HMS01490[g]	lethal			
Negative	Spinophilin	Spn	CG16757	19658 (S)	4%	56		
				105888	1%	106		
Negative	sprite	sprt	CG30023	107873	2%	122		
Negative	SRY interacting protein 1	Sip1	CG10939	16958	15%	65	9%	170
Negative	still life	sif	CG34418	26132 (M)	7%	89		
				106832	1%	106		
Negative	Syntrophin-like 1	Syn1	CG7152	27893	4%	73		
				104992	3%	111		
				JF02654[g]	3%	138		
Negative	varicose	vari	CG9326	24156 (M†)	4%	70		
				104548	6%	87		
				HM05087[g]	2%	124		
Negative	X11L	X11L	CG5675	28652	9%	110		
				27479	9%	114		
Negative	X11Lbeta	X11Lbeta	CG32677	8309 (S,M†)	2%	51		
Negative	Z band alternatively spliced PDZ-motif protein 66	Zasp66	CG6416	102980	5%	131		
Not tested	arc	a	CG6741					
Not tested	Myosin heavy chain-like	Mhcl	CG31045					

[a] Group designation are as follows: Pos. Mult. = Positive, Multiple Hits; Pos. Single = Positive, Single Hit; Negative = No Hit.
[b] Percentage of stage 10 egg chambers in which border cell migration defects were observed upon RNAi expression.
[c] Number of stage 10 egg chambers scored per trial. N ≥ 50 in every trial.

[d] Lines that produced a phenotype (M) or were lethal (M†) in a genome-wide RNAi screen to study Notch signaling in Drosophila (Mummery-Widmer et al., 2009).
[e] Values highlighted in orange represent a migration defect (≥10%).
[f] Lines that produced a phenotype (S) or were lethal (S†) in a genome-wide RNAi screen to study Drosophila muscle morphogenesis and function (Schnorrer et al., 2010).
[g] RNAi lines from the Transgenic RNAi Project (TRiP), Harvard Medical School.
[h] LIMK1 double RNAi transgenic line (Ng insertions. Personal communication to FlyBase [FBrf0188570]).
[i] par-6 double RNAi transgenic line from Pinheiro and Montell, 2004.
[j] RNAi lines from NIG-Fly, Japan.
[k] RhoGAP19D RNAi transgenic lines (Luo insertions. Personal communication to FlyBase [FBrf0141849]).
