# Supplementary material for: On the Role of PDZ Domain-Encoding Genes in Drosophila Border Cell Migration
Source: G3 (Bethesda). 2012 Nov 1;2(11):1379–91. doi: 10.1534/g3.112.004093 (PMC3484668; doi:10.1534/g3.112.004093)
Supplement: Supporting Information [file supp_2.11.1379_FigureS1.pdf]

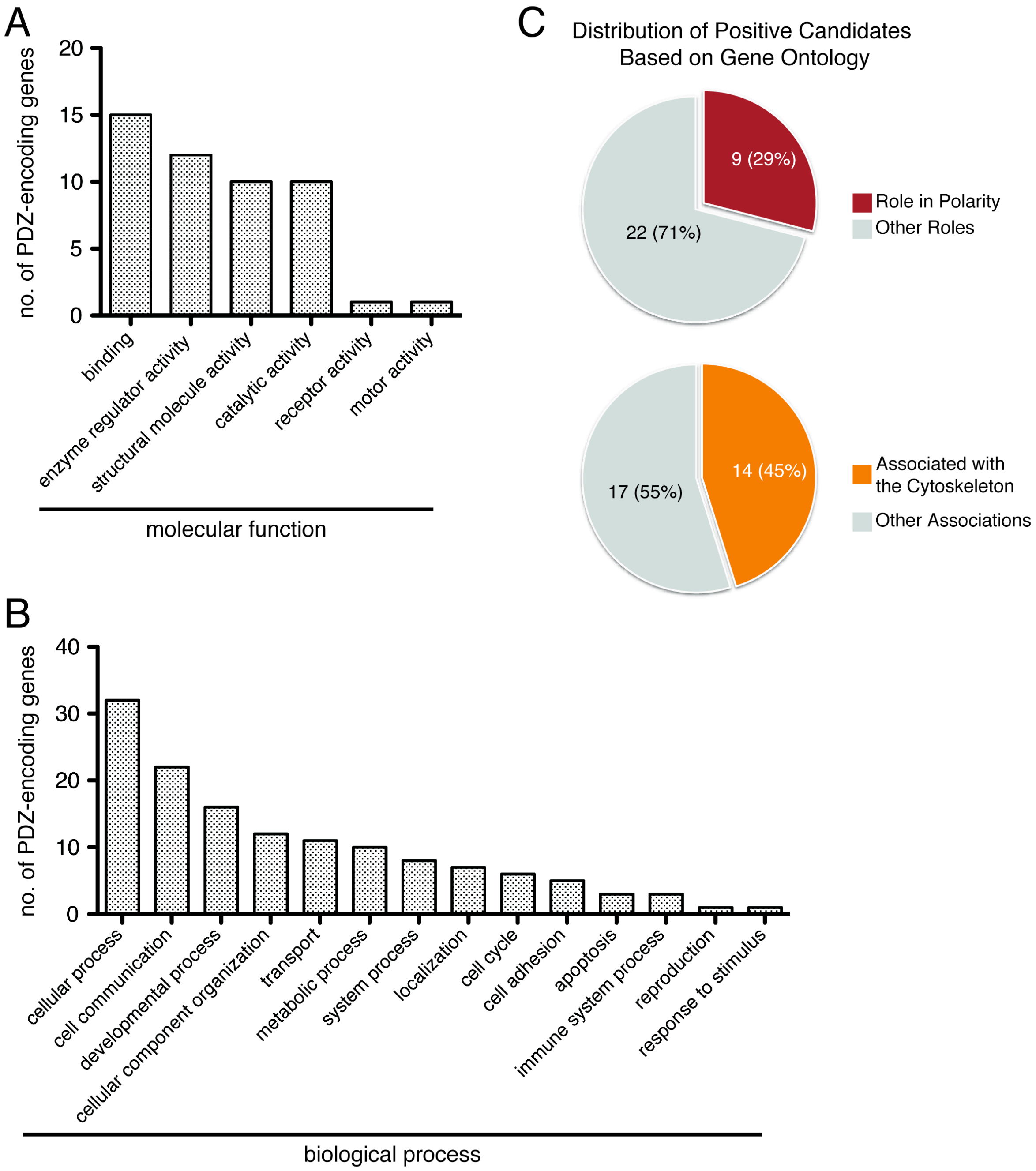

Figure S1 Drosophila PDZ-domain-containing proteins are functionally diverse and involved in numerous processes. (A-B) Of the 66 genes annotated to contain a PDZ domain, 51 were analyzed by PANTHER (http://www.pantherdb.org). (A) PDZ proteins are found in almost half (6/14) of the classifications for molecular function and predominantly annotated as having binding, enzyme regulator, structural, or catalytic activity. Molecular function is defined as the action that a protein performs on its direct target. A protein is assigned only one molecular function. (B) PDZ proteins contribute to 14 out of 19 annotated biological processes. A protein can contribute to multiple processes. (C) Positive hit genes were surveyed for roles in polarity or association with the cytoskeleton using AmiGO (http://amigo.geneontology.org). Genes can fall into both categories. The polarity genes include: *baz, CASK, dsh, Lap1, par-6, Patj, pyd, sdt* and *veli*. The following genes are associated with the cytoskeleton: *CG14168, CG42319, CG42788, CG5921, CG6498, dsh, Lap1, LIMK1, Patj, Ptpmeg, RhoGAP19D, Syn2, veli,* and *Zasp52*.
