# Supplementary material for: On the Role of PDZ Domain-Encoding Genes in Drosophila Border Cell Migration
Source: G3 (Bethesda). 2012 Nov 1;2(11):1379–91. doi: 10.1534/g3.112.004093 (PMC3484668; doi:10.1534/g3.112.004093)
Supplement: Supporting Information [file supp_2.11.1379_FigureS2.pdf]

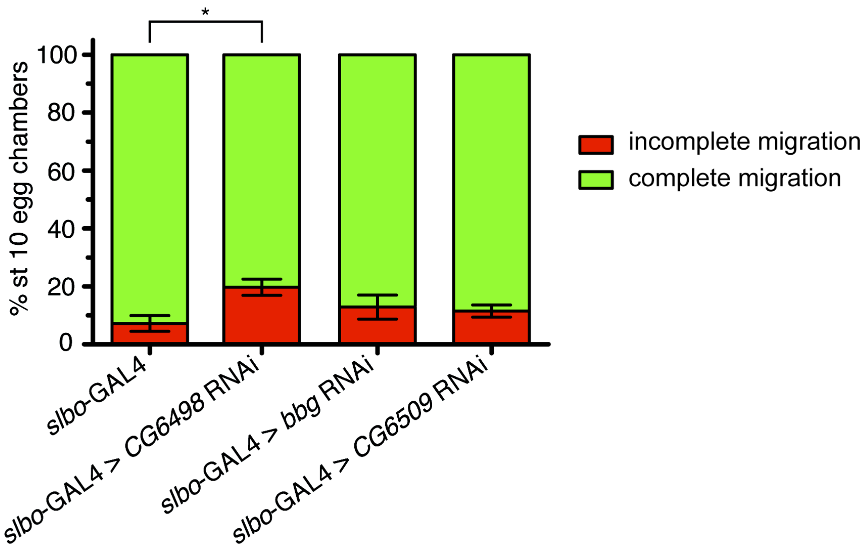

Figure S2 *slbo*-GAL4-driven RNAi expression targeting three positive candidates. Quantification of border cell migration at stage 10, shown as the percentage of border cells with complete (green) or incomplete (red) migration in egg chambers expressing *CG6498* RNAi v35100, *bbg* RNAi v15975, and *CG6509* RNAi v22496 in border cells using *slbo*-GAL4. Two-tailed unpaired t-test was used to determine statistical significance (*, $p < 0.05$). At least 50 egg chambers were scored in at least three trials.
