# Supplementary material for: On the Role of PDZ Domain-Encoding Genes in Drosophila Border Cell Migration
Source: G3 (Bethesda). 2012 Nov 1;2(11):1379–91. doi: 10.1534/g3.112.004093 (PMC3484668; doi:10.1534/g3.112.004093)
Supplement: Supporting Information [file supp_2.11.1379_FigureS3.pdf]

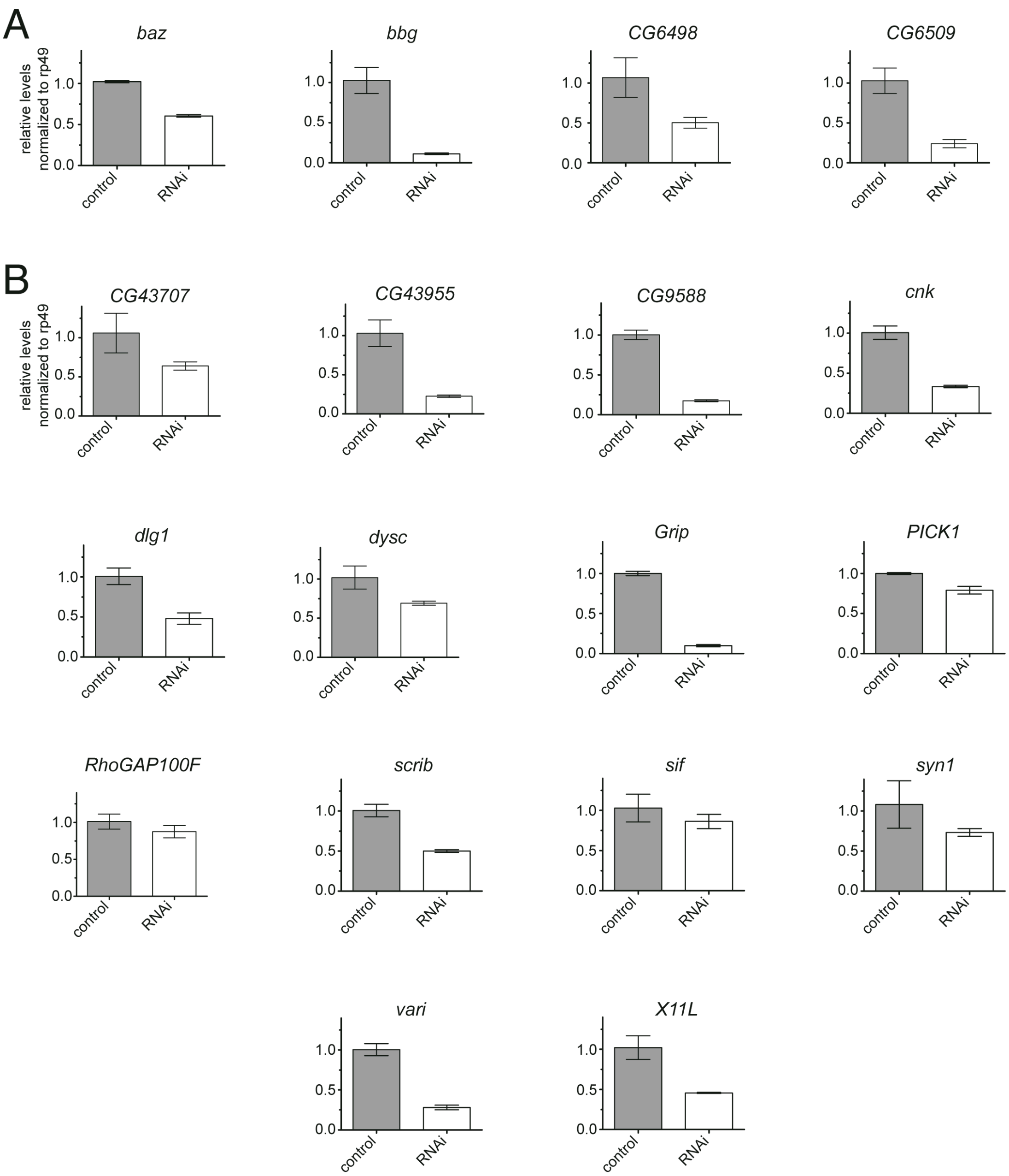

Figure S3 Gene expression levels in control and RNAi knockdown measured by quantitative RT-PCR. Ubiquitous expression of RNAi against a subset of (A) positive candidates and (B) negative candidates result in varying degrees of knockdown. The mean transcript levels were calculated from three biological replicates using $\Delta\Delta C_T$ method and normalized to *rp49* mRNA levels. Controls are RNAi to GFP. Error bars represent standard deviation.
