# Supplementary material for: On the Role of PDZ Domain-Encoding Genes in Drosophila Border Cell Migration
Source: G3 (Bethesda). 2012 Nov 1;2(11):1379–91. doi: 10.1534/g3.112.004093 (PMC3484668; doi:10.1534/g3.112.004093)
Supplement: Supporting Information [file supp_2.11.1379_FigureS4.pdf]

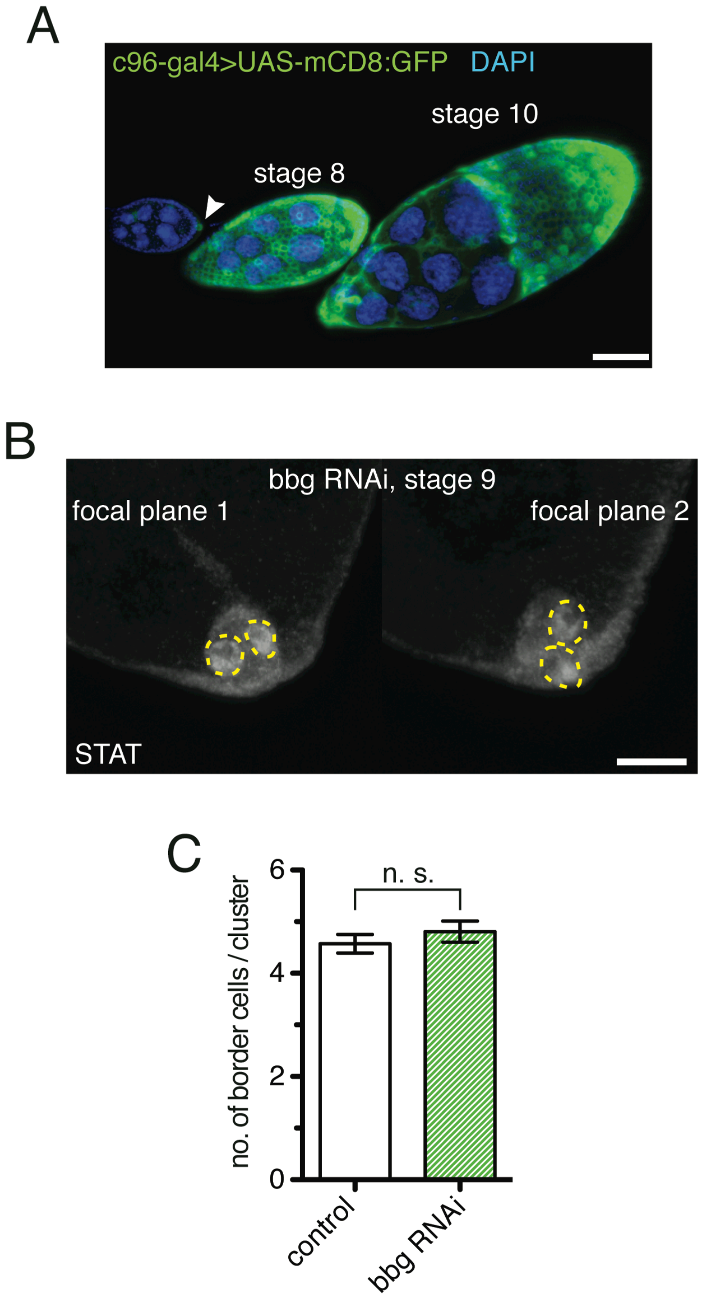

Figure S4 Bbg function in early development of border cells. (A) *c96*-GAL4 expression (green) in egg chambers is limited to the polar cells (arrowhead) in the early stages but expands to most of the follicle cell epithelium starting at stage 8 of Drosophila oogenesis. The image was acquired to show the follicle cell epithelium. The nuclei are visualized with DAPI (blue). Scale bar is 50 μm. (B) STAT nuclear enrichment (yellow dashed circles) is observed in border cells expressing *bbg* RNAi line v15975 driven by *c306*-GAL4 before detachment (stage 9). Upon *bbg* knockdown, 72% (N=21) of detaching clusters still retain nuclear STAT enrichment in all or some border cells in the cluster. The images are of the same border cell cluster taken at different focal planes. Scale bar is 10 μm. (C) Border cell recruitment is unaffected by *bbg* RNAi. On average, both control (N=35) and bbg RNAi (N=31) clusters have ~5 border cells. Error bars represent SEM. N.S., not statistically significant (two-tailed unpaired t-test).
