# Supplementary material for: On the Role of PDZ Domain-Encoding Genes in Drosophila Border Cell Migration
Source: G3 (Bethesda). 2012 Nov 1;2(11):1379–91. doi: 10.1534/g3.112.004093 (PMC3484668; doi:10.1534/g3.112.004093)
Supplement: Supporting Information [file supp_2.11.1379_TableS1.pdf]

Table S1 Complete results of the PDZ RNAi survey of border cell migration

Group[a]	Gene Name	Gene Symbol	Annotation Symbol	RNAi line	trial 1		trial 2		trial 3	
					% defect[b]	n[c]	% defect	n	% defect	n
Pos. Mult.	bazooka	baz	CG5055	2914 (M)[d]	43%[e]	116				
				2915 (M)	23%	137				
Pos. Mult.	big bang	bbg	CG42230	15975	19%	58	15%	112		
				101691	10%	132				
				36111	6%	78				
Pos. Mult.	CASK ortholog	CASK	CG6703	34185 (S†,M)[f]	23%	79	15%	80		
				34184	22%	122	10%	251		
				104793	2%	139				
Pos. Mult.	CG5921	CG5921	CG5921	37875	33%	94	23%	106		
				37874	18%	91				
Pos. Mult.	CG6498	CG6498	CG6498	35101	20%	97	11%	125		
				35100	17%	116	12%	164		
				109282	13%	176				
Pos. Mult.	CG6509	CG6509	CG6509	46234 (S†,M)	11%	54	12%	139		
				101596	11%	74				
				22496 (S†,M)	9%	87				
Pos. Mult.	Gef26	Gef26	CG9491	27017 (M)	19%	86	28%	197		
				27015 (M)	10%	98	20%	98		
				105159	15%	59				
Pos. Mult.	Lap1	Lap1	CG10255	18600 (M)	15%	112	13%	119		
				JF02362[g]	10%	134				
Pos. Mult.	LIM-kinase1	LIMK1	CG1848	25344	10%	125	8%	194	12%	220
				25343	6%	128				
				2x RNAi[h]	18%	50				
Pos. Mult.	par-6	par-6	CG5884	19732 (M†)	38%	58	26%	114		
				108560	44%	59				
				2x RNAi[i]	14%	132				
Pos. Mult.	PatJ	Patj	CG12021	31620	13%	168	20%	128		
				12021R-3[j]	26%	184				
				101877	3%	63				
				12021R-4[j]	lethal		lethal			
Pos. Mult.	Rim	Rim	CG33547	39384	26%	92	12%	50		
				39385	24%	94	1%	78	12%	113
				JF02610[g]	7%	254				
Pos. Mult.	stardust	sdt	CG32717	100685	39%	62	11%	57		
				29844	13%	182				
				29843	6%	197				
Pos. Mult.	veli	veli	CG7662	43094 (M†)	50%	88	27%	101		

				46963 (M)	31%	61				
Pos. Single	CG14168	CG14168	CG14168	17414	13%	55	21%	226		
				103225	2%	56				
Pos. Single	CG34375	CG34375	CG34375	23904	29%	63	5%	166	13%	143
				102586	4%	56				
Pos. Single	CG42319	CG42319	CG42319	23928	41%	152				
				32835 (M)	8%	197				
Pos. Single	CG42788	CG42788	CG42788	22311	40%	72	5%	134	11%	196
				108180	1%	123				
				45034	4%	72				
Pos. Single	CG6688	CG6688	CG6688	48260	15%	86	19%	214		
				106732	1%	139				
Pos. Single	Dishevelled	dsh	CG18361	101525	12%	93				
				JF01254[g]	6%	188				
				18361R-2[j]	lethal					
				18361R-3[j]	lethal					
				JF01253[g]	8%	89				
Pos. Single	Efa6 pleckstrin and Sec7 domain containing	Efa6	CG31158	42321	14%	128	12%	109		
Pos. Single	HtrA2	HtrA2	CG8464	24104	16%	75	9%	153	19%	260
				24106	9%	132				
	Magi	Magi	CG30388	41735	13%	128	4%	167		
				41736	10%	62				
Pos. Single	menage a trois	metro	CG30021	29967	10%	73				
				29965	1%	128				
				110814	1%	129				
Pos. Single	polychaetoid	pyd	CG43140	38863	15%	111	23%	188		
				104159	6%	142				
Pos. Single	Ptpmeg	Ptpmeg	CG1228	38651	12%	165	22%	78		
				103740	3%	130				
Pos. Single	RhoGAP19D	RhoGAP19D	CG1412	43955 (M†)	42%	57	59%	165		
				HMS00352[g]	9%	161				
				dsRNA 2.1[k]	8%	217				
				dsRNA 3.1[k]	3%	62				
Pos. Single	Rhophilin	Rhp	CG8497	24111	13%	102	11%	204		
				110377	3%	152				
Pos. Single	Slip1	Slip1	CG1783	33006 (S)	22%	124	10%	168		
				101106	6%	71				
Pos. Single	Syntrophin-like 2	Syn2	CG4905	JF02999[g]	11%	157	6%	84		
				110602	10%	67				
Pos. Single	Z band	Zasp52	CG30084	36563 (S†,M†)	37%	97	19%	124		

	alternatively spliced PDZ-motif protein 52							
				106177	3%	65		
				JF01133[g]	4%	227		
Negative	canoe	cno	CG42312	102686	3%	92		
Negative	CG10362	CG10362	CG10362	8317	4%	52		
Negative	CG15617	CG15617	CG15617	19148	37%	65	7%	162
				19149	7%	94	6%	167
				103267	2%	50		
Negative	CG15803	CG15803	CG15803	43635	3%	59		
				43636	1%	135		
Negative	CG32758	CG32758	CG32758	28457	4%	99		
				108542	4%	130		
Negative	CG3402	CG3402	CG3402	21485 (M†)	7%	58		
				110191	6%	125		
Negative	CG43707	CG43707	CG43707	25846S	8%	130		
				25847 (S†)	6%	89		
Negative	CG43955	CG43955	CG43955	103267	2%	50		
Negative	CG6619	CG6619	CG6619	15622	4%	67		
Negative	CG9588	CG9588	CG9588	47763	2%	95		
				100126	4%	167		
				HM05013[g]	0%	93		
Negative	connector enhancer of kar	cnk	CG6556	107746	3%	135		
				HMS00238[g]	3%	152		
Negative	discs large 1	dlg1	CG1725	41134 (S†,M†)	4%	85		
				109274	2%	132		
				41136	lethal			
				HMS01521[g]	lethal			
Negative	dyschronic	dysc	CG43749	23278	9%	92		
				14082	5%	59		
				110019	3%	135		
Negative	Glutamate receptor binding protein	Grip	CG14447	21003 (S†)	5%	57		
				103551	6%	71		
Negative	Grasp65	Grasp65	CG7809	22564	1%	125		
Negative	inactivation no afterpotential D	inaD	CG3504	26211	6%	64		
Negative	kermit	kermit	CG11546	109297	2%	131		
Negative	locomotion defects	loco	CG5248	9248 (M)	10%	67	6%	237

				110275	7%	149		
				HMS00455[g]	3%	189		
Negative	PICK1	PICK1	CG6167	22268	4%	74		
				104486	1%	163		
				JF01199[g]	3%	222		
Negative	Prosap	Prosap	CG30483	21216 (M†)	5%	59		
				103592	4%	98		
Negative	Protostome-specific GEF	PsGEF	CG43947	32088	8%	122		
				109769	1%	154		
Negative	RhoGAP100F	RhoGAP100F	CG1976	106241	0%	62		
				HMS00740[g]	1%	161		
Negative	RhoGEF2	RhoGEF2	CG9635	JF01747[g]	13%	145	6%	135
				HMS01118[g]	7%	180		
				110577	3%	90		
Negative	scribbled	scrib	CG43398	105412	5%	172		
				HMS01490[g]	lethal			
Negative	Spinophilin	Spn	CG16757	19658 (S)	4%	56		
				105888	1%	106		
Negative	sprite	sprt	CG30023	107873	2%	122		
Negative	SRY interacting protein 1	Sip1	CG10939	16958	15%	65	9%	170
Negative	still life	sif	CG34418	26132 (M)	7%	89		
				106832	1%	106		
Negative	Syntrophin-like 1	Syn1	CG7152	27893	4%	73		
				104992	3%	111		
				JF02654[g]	3%	138		
Negative	varicose	vari	CG9326	24156 (M†)	4%	70		
				104548	6%	87		
				HM05087[g]	2%	124		
Negative	X11L	X11L	CG5675	28652	9%	110		
				27479	9%	114		
Negative	X11Lbeta	X11Lbeta	CG32677	8309 (S,M†)	2%	51		
Negative	Z band alternatively spliced PDZ-motif protein 66	Zasp66	CG6416	102980	5%	131		
Not tested	arc	a	CG6741					
Not tested	Myosin heavy chain-like	Mhcl	CG31045					

[a] Group designation are as follows: Pos. Mult. = Positive, Multiple Hits; Pos. Single = Positive, Single Hit; Negative = No Hit.
[b] Percentage of stage 10 egg chambers in which border cell migration defects were observed upon RNAi expression.
[c] Number of stage 10 egg chambers scored per trial. N ≥ 50 in every trial.

[d] Lines that produced a phenotype (M) or were lethal (M†) in a genome-wide RNAi screen to study Notch signaling in Drosophila (Mummery-Widmer et al., 2009).
[e] Values highlighted in orange represent a migration defect (≥10%).
[f] Lines that produced a phenotype (S) or were lethal (S†) in a genome-wide RNAi screen to study Drosophila muscle morphogenesis and function (Schnorrer et al., 2010).
[g] RNAi lines from the Transgenic RNAi Project (TRiP), Harvard Medical School.
[h] LIMK1 double RNAi transgenic line (Ng insertions. Personal communication to FlyBase [FBrf0188570]).
[i] par-6 double RNAi transgenic line from Pinheiro and Montell, 2004.
[j] RNAi lines from NIG-Fly, Japan.
[k] RhoGAP19D RNAi transgenic lines (Luo insertions. Personal communication to FlyBase [FBrf0141849]).
